# Tracking market and non-traditional sources of risks in procyclical and countercyclical hedge fund strategies under extreme scenarios: a nonlinear VAR approach

**DOI:** 10.1186/s40854-021-00316-3

**Published:** 2022-03-07

**Authors:** François-Éric Racicot, Raymond Théoret

**Affiliations:** 1 Telfer School of Management, University of Ottawa, 55 Laurier Avenue East, Ottawa, ON Canada; 2 IPAG Business School, Paris, France; 3 École des Sciences de la Gestion, Université du Québec (Montréal), 315 est Ste-Catherine, R2915, Montréal, QC Canada; 4 Chaire d’Information Financière et Organisationnelle, ESG-UQAM, Monntréal, Québec, Université du Québec (Outaouais), Montréal, Canada

**Keywords:** Hedge fund, Procyclicality, Illiquidity risk shock, Illiquidity uncertainty shock, Local projection model, TVAR, Optimal forecast, Measurement errors, C13, C58, G11, G23

## Abstract

The subprime crisis was quite damaging for hedge funds. Using the local projection method (Jordà 2004, 2005, 2009), we forecast the dynamic responses of the betas of hedge fund strategies to macroeconomic and financial shocks—especially volatility and illiquidity shocks—over the subprime crisis in order to investigate their market timing activities. In a robustness check, using TVAR (Balke 2000), we simulate the reaction of hedge fund strategies’ betas in extreme scenarios allowing moderate and strong adverse shocks. Our results show that the behavior of hedge fund strategies regarding the monitoring of systematic risk is highly nonlinear in extreme scenarios—especially during the subprime crisis. We find that countercyclical strategies have an investment technology which differs from procyclical ones. During crises, the former seek to capture non-traditional risk premia by deliberately increasing their systematic risk while the later focus more on minimizing risk. Our results suggest that the hedge fund strategies’ betas respond more to illiquidity uncertainty than to illiquidity risk during crises. We find that illiquidity and VIX shocks are the major drivers of systemic risk in the hedge fund industry.

## Introduction

Hedge fund strategies should be studied in a dynamic setting (Fung and Hsieh 1997, 2001, 2004). Indeed, their transactions are designed to be nonlinear or highly nonlinear with respect to the underlying assets. Since these transactions are option-like, the dynamic dimension was initially introduced in hedge-fund asset pricing models by resorting to the prices of standard options (long or short puts) or to more complex structured products (e.g., lookback straddles) as additional factors in conventional asset pricing models (Fung and Hsieh 1997, 2001, 2004; Mitchell and Pulvino 2001; Agarwal and Naik 2004). These options span various forms of market timing by portfolio managers (Glosten and Jagannathan 1994; Agarwal and Naik 2004; Stafylas et al. 2017). The exposure of hedge-fund returns to risk factors, especially the market risk premium, has been made time-varying relatively recently, particularly since the subprime crisis (Holmes and Faff 2008; Billio et al. 2012; Jawadi and Khanniche 2012; Bali et al. 2014; Namvar et al. 2016; Stafylas et al. 2017; Racicot and Théoret 2016; Thomson and van Vuuren 2018; Lambert and Platania 2016, 2020; Racicot et al. 2021). The literature indicates that hedge funds monitor their risk exposure in order to take more risk in high regimes (i.e., expansions) and less risk in low regimes (i.e., recessions). Consistent with this finding, Calluzzo et al. (2019) contend that hedge funds are associated with “smart money” in the sense that they attenuate market anomalies. Hence, hedge funds seem to be good market timers and arbitrageurs. Hedge-fund strategies could even aim at “decorrelating” their transactions from financial markets when they are bearish with the help of short sales, fire sales, hedging operations, and/or deleveraging (Brunnermeier and Pedersen 2009; Shleifer and Vishny 2010; Brunnermeier and Sannikov 2014). Thus, it appears that hedge fund strategies may succeed quite well in monitoring their exposure to shocks, especially by modifying their betas, in order to optimize their risk-return trade-off in high and low regimes. However, the 2008 subprime crisis was particularly stressful for hedge funds. In fact, the assets under management in the hedge fund industry fell by nearly 30% (i.e., US$ 1.5 trillion) in 2008, the largest decline on record (Saunders et al. 2014).^1^

This study tracks the risk monitoring of hedge fund strategies during bad times, featuring a local projection (LP) over the course of the subprime crisis, the worst crisis since the Great Depression, in order to compare data on how hedge funds responded to shocks during the crisis to results obtained with a standard linear Vector Autoregressive (VAR) process computed over the whole sample period, reflecting the “average” behavior of the strategies. Impulse response functions (IRF) computed with the LP technique are preferable to the standard VAR when the data-generating process is nonlinear because they are based on a direct forecast (Jordà 2004, 2005).

The first major contribution of this study is its use of a dynamic approach to examine a database of hedge fund strategies spanning 1988–2016 to better assess their resilience to adverse shocks. To the best of our knowledge, this study is the first to adopt this framework to investigate hedge fund strategies’ market timing. Most studies that explore hedge fund market timing adopt a static approach, relying on cross-sectional-panel regressions and quantile analyses rather than a multivariate dynamic time series approach (Fung and Hsieh 1997, 2001, 2004; Getmansky et al. 2004; Bali et al 2014; Agarwal et al. 2017). Moreover, these studies neglect to model the dynamic (time-dependent) nature of market timing or the time-varying correlation between endogenous variables. This can be performed properly only through a dynamic VAR setting based on time-series forecasts rather than on cross-sections. As shown in our empirical experiments, a static approach may not reveal significant responses of the conditional beta to shocks, whereas our VAR approach may capture complex dynamic responses to them. Our illiquidity experiments are particularly instructive on this point. Overcoming the limitations of the literature’s approach to market timing is the main motivation for this study.^2^

Before running our VAR experiments, we use a simple device borrowed from Stiroh (2004) to examine whether the beta of a hedge fund strategy is procyclical (i.e., responds positively to GDP growth) or countercyclical (i.e., responds negatively to GDP growth). This exercise is important because our estimations show that countercyclical strategies have an investment technology different from that of procyclical strategies. During recessions or crises, the former seeks to capture risk premia (spreads), which are associated with volatility risk, credit risk, and illiquidity risk (i.e., non-traditional sources of risk that may attract investors), while the latter focuses more on minimizing risk. In other words, the cyclicality of the hedge fund strategies’ beta is instrumental in their responses to various shocks. Apart from the general index, we find that the most procyclical strategies are the growth, event-driven, multistrategy, macro, and value index types. These are also the most likely to respond to external shocks, such as macroeconomic and financial shocks. The strategies most related to bond markets, or that follow contrarian activities, are countercyclical: short sellers, distressed, fixed income, convertibles, long-short credit, and futures.

Our LP experiments during the subprime crisis show that the negative response of the betas of many strategies to an adverse GDP growth shock is significantly higher during the crisis than during normal times, as measured by the benchmark linear VAR, especially for short sellers, value index, growth, long-short credit, mergers, opportunity index, convertibles, and multistrategy. Asymmetries are much greater for a VIX shock than for a GDP growth shock in the sense that hedge fund strategies’ betas respond much more strongly during crises than during normal times. The response of the procyclical strategies’ betas is also important during the subprime crisis: The reaction obtained with the LP procedure is greater than that obtained with the standard benchmark VAR. We also find that the beta of contrarian strategies increased during the subprime crisis and that their behavior was more asymmetric than that of the other strategies. Our LP model also includes the credit spread and term spread shocks. The betas of the strategies that embed a lot of credit risk—that is, bond-oriented strategies such as convertibles, fixed income, and long-short credit—react the most strongly following a credit spread shock. Interestingly, the beta of the distressed securities strategy increases after a credit spread shock, which suggests that investing in high-yield bonds benefits from this kind of shock when the probability of corporate default peaks. Finally, the beta of strategies holding the most liquid assets under management—such as futures, short-sellers, and growth—decreases following a term spread shock, which stands for an illiquidity shock in our setting. For both credit spread and term spread shocks, we find that the strategies’ response is low when using the benchmark linear VAR, which is a clear indication of asymmetry.

The second major contribution of this study is its experiment with the measure of illiquidity risk, conducted to better grasp the specific impact of illiquidity shocks. Our measure, “innovation in aggregate liquidity,”^3^ was developed by Pástor and Stambaugh (2003) and is usually employed as a risk factor in cross-sectional (static) regressions. In line with the methodology developed by Beaudry et al. (2001) to build macroeconomic uncertainty measures, we devise a new indicator of illiquidity uncertainty, the conditional variance of the innovation in aggregate liquidity, which closely tracks crisis periods. Using our nonlinear VAR approaches, we find that the conditional beta of hedge fund strategies may respond more to illiquidity uncertainty than to illiquidity risk, a new finding in the hedge fund literature. Interestingly, since our measure of liquidity uncertainty is a generated variable, it is subject to a measurement error bias that might lead to inconsistent estimators (Pagan 1984, 1986; Pagan and Ullah 1988). We correct for this bias using innovative robust instruments (Fuller 1987; Lewbel 1997; Racicot 2015; Racicot and Rentz 2015; Racicot et al. 2018; Racicot et al. 2019) before introducing our indicator in LP. We show that neglecting this bias may lead to spurious results. Moreover, our forecasts reveal that strategies may have a complex reaction to illiquidity shocks: Some strategies may capture the illiquidity premium with prudence,^4^ while other strategies may do so more aggressively. To the best of our knowledge, we are the first to adopt such a framework to analyze the impact of well-known measures of illiquidity in the hedge industry.

As a robustness check, we use a nonlinear VAR, the threshold VAR (TVAR, Balke 2000), which allows us to analyze the impact of moderate and strong positive and negative shocks over two regimes optimally defined: a high (expansion) and low (recession or crisis) regime. We conjecture that strong adverse shocks are more difficult to control than moderate ones. We must rely on a few variables when using this method because it consumes many degrees of freedom.^5^ Thus, we consider two important kinds of shocks to run the TVAR: (i) a GDP growth shock (macroeconomic or business cycle shock) and (ii) a VIX shock (financial shock), the VIX being an indicator of the implicit volatility of U.S. stock markets but also an investor’s fear gauge (Diebold and Yilmaz 2014). We also conduct our experiments using our illiquidity measures. Balke’s procedure allows the study of two types of nonlinearities: (i) asymmetry—that is, a different reaction in high and low regimes; and (ii) non-proportionality—that is, the reaction to positive or negative shocks, or to moderate and strong shocks, that may be non-proportional. Asymmetry is present in our experiments because the amplitude of the strategies’ IRFs is usually greater in the low than in the high regime for both GDP growth and VIX shocks.

Our findings using the TVAR complement the results obtained using the LP method. We find that the representative hedge fund may struggle to control a strong negative GDP shock at its impact, whereas this is much less true for a moderate one—an obvious case of non-proportionality.^6^ Moreover, some countercyclical strategies—convertibles, distressed, fixed income, and long-short credit—display a reaction to GDP growth shocks that is quite different from those of procyclical strategies. Indeed, their beta increases in a nonlinear way^7^ following a negative shock, which might be explained by their attempt to capture the risk premia or spreads related to their activities. Similar to the finding of the LP method, we find that the beta of hedge fund strategies is more sensitive to financial shocks (i.e., VIX shocks) than to business cycle shocks (i.e., GDP growth shocks; Stafylas et al. 2018). We note that strategies embedded with the highest betas—growth, value index, opportunity index, event-driven, and multistrategy—are the most responsive to VIX shocks. Their response is much more important in the low regime, an obvious case of asymmetry.

The remainder of this paper is organized as follows. “Background literature” Section provides background literature related to market timing. “Data and stylized facts” Section presents the study’s database and stylized facts. “Empirical results” section introduces the LP method and provides the main empirical results. “Robustness check: response of hedge fund strategies to illiquidity shocks” section analyses the impact of well-known measures of illiquidity on the returns and conditional betas of hedge fund strategies using pooled regressions with SUR defined over crises and local projections. “Policy implications” section presents the policy implications of our study, while “Conclusion” section concludes the paper. “Appendix 1” analyzes the responses of strategies’ betas to macroeconomic and financial shocks using the TVAR, while “Appendix 2” focuses on the impact of another well-known illiquidity indicator, the Amihud ratio.

## Background literature

We outline the motivation of our study below by briefly surveying the background research.

Studies aimed at analyzing the market-timing of the portfolio beta—especially in the hedge fund industry—may be divided into two categories: (i) studies that tend to rely on “static” transformations or extensions of standard asset pricing models (e.g., the CAPM, the Fama and French model, or regression-based style analysis; Sharpe 1992) to track the market-timing behavior of funds regarding their risk; and (ii) studies that use the tools of dynamic econometrics (e.g., the Kalman filter or VAR) to analyze the monitoring of systematic risk by hedge funds. Most of these studies have been performed after the subprime crisis.^8^

### Static approaches to market timing

The first set of studies on beta market timing begins with Treynor and Mazuy (1966), who introduce a quadratic term in the CAPM—the square of the market risk premium—to analyze the market timing performance of mutual funds. They write their model as follows: $R_{pt} - R_{ft} = \alpha_{p} + \gamma_{1} \left( {R_{mt} - R_{ft} } \right) + \gamma_{2} \left( {R_{mt} - R_{ft} } \right)^{2} + \varepsilon_{t}$, where $R_{pt}$ is the portfolio return, $R_{ft}$ is the risk-free interest rate, $\alpha_{p}$ is the alpha of Jensen, $R_{mt}$ is the market portfolio return, and $\varepsilon_{t}$ is innovation. If the relationship between the portfolio excess return and the market excess return is convex—that is, if $\gamma_{2}$ is positive—this signals good market timing because the beta is higher in bullish stock markets. Later, Henrisksson and Merton (HM) (1981) rely on an alternative transformation of the CAPM to test market timing—that is, $R_{pt} - R_{ft} = \alpha_{p} + \pi_{1} \left( {R_{mt} - R_{ft} } \right) + \pi_{2} \left( {R_{mt} - R_{ft} } \right)D + \varepsilon_{t}$, where *D* is a dummy variable taking the value of one if $R_{mt} > R_{ft}$ (i.e., in bullish markets) and zero otherwise (i.e., in bearish markets). Good market timing is associated with a positive sign for $\pi_{2}$ since the beta is then higher when stock markets trend upward, which is the essence of good market timing, with portfolio managers taking more risk to capture positive payoffs. The HM equation can be rewritten as

$$R_{pt} - R_{ft} = \alpha_{p} + \pi_{1} \left( {R_{mt} - R_{ft} } \right) + \pi_{2} MAX\left( {R_{mt} - R_{ft} ,0} \right) + \varepsilon_{t}$$

Good market timing thus amounts to a call option on the market portfolio return (Glosten and Jagannathan 1994; Agarwal and Naik 2004; Stafylas et al. 2017). Despite its simplicity, the HM equation paved the way for studies on market timing performed by relying on the option approach. However, these earlier methods are still simple ways to capture unobservable time-varying beta. Ferson and Schadt (1996) write a functional form for the unobservable time-varying beta, which is then incorporated into a conventional asset pricing model. For example, assume a CAPM model in which beta is time-varying—thus, $R_{pt} - R_{mt} = \alpha_{p} + \beta_{pt} \left( {R_{mt} - R_{ft} } \right) + \varepsilon_{t}$. To estimate $\beta_{pt}$, write a functional form for it—that is, $\beta_{pt} = c + \theta x_{{_{t} }}$, where *x*_*t*_ is a macroeconomic or financial variable that is assumed to rule market timing. Replacing $\beta_{pt}$ by its functional expression in the CAPM model, we obtain

1 $$R_{pt} - R_{ft} = \alpha_{p} + c\left( {R_{mt} - R_{ft} } \right) + \theta \left( {R_{mt} - R_{ft} } \right)x_{t} + \varepsilon_{t}$$

The parameters of the beta equation may then be easily estimated, and we can thus compute a time series that proxies for the unobserved time-varying beta—that is, $\hat{\beta }_{pt} = \hat{c} + \hat{\theta }x_{t}$. This method is a precursor to the use of the Kalman filter for estimating the time-varying beta, which then becomes a state or unobserved variable (Racicot et al. 2019).^9^ Note that, if $x_{t} = R_{mt} - R_{ft}$ in Eq. (1), we consider the model of Treynor and Mazuy (1966). This latter model may thus be viewed as a particular case of Ferson and Schadt’s model (1996).

The option approach to the analysis of market timing in the hedge fund industry is indebted mainly to Fung and Hsieh (1997, 2001, 2002, 2004) and Mitchell and Pulvino (2001). For these authors, hedge fund transactions are essentially option-like and thus dynamic. For Mitchell and Pulvino (2001), many hedge funds display payoffs that are similar to a short put. Thus, these funds are short volatility. By contrast, the payoffs of the trend followers and market timers (e.g., CTA) may be associated with the payoffs of lookback straddles (Fung and Hsieh 1997, 2001, 2002, 2004; Stafylas et al. 2017). In this respect, the payoffs of a perfect market timer who is not involved in short sales are similar to those of a long call option, as suggested by the HM equation. When hedge funds are involved in short sales—a current practice in the hedge fund industry—perfect market timing then delivers payoffs similar to those of lookback straddles (Fung and Hsieh 2004; Stafylas et al. 2017).

### Dynamic econometric approaches to market timing

The subprime crisis, from the middle of 2007 to the end of 2009, can serve as a laboratory for studying the market timing activities of hedge funds because of its severity. Studies conducted after the crisis rely on sophisticated econometric dynamics techniques to monitor hedge fund risk management. Researchers are concerned about the asymmetric behavior of hedge funds depending on the phase of the business cycle (Holmes and Faff 2008; Jawadi and Khanniche 2012; Namvar et al. 2016; Stafylas et al. 2018). The Kalman filter is a tool used in a large number of studies (Thomson and van Vuuren 2018; Lambert and Platania 2020).^10^ Other dynamic econometric techniques have also been used. For instance, Stafylas et al. (2018) resort to a Markov-switching approach. They find that hedge fund managers tend to reduce their systematic risk during bad times, whereas they increase their risk-taking during good times to deliver abnormal returns (alphas). This result is in line with the findings of Holmes and Faff (2008), who devised their time-varying betas with the Kalman filter. They indicate that hedge funds decrease their beta when market volatility increases; that is, they are short volatility when market fear as gauged by market volatility is on an upward trend, rather than increase their exposure to volatility. This result supports the finding of Mitchell and Pulvino (2001), who assert that the payoffs of numerous hedge funds behave as short puts. Since hedge funds make an intensive use of derivatives, one would expect that they would have exposed themselves to an increase in stock market volatility by being long volatility. Their opposite reaction seems to be at odds with the vocation of hedge funds since a long position in conventional options benefits from an increase in volatility. We may conclude that the managers of many hedge funds are risk-averse. Racicot and Théoret (2018) rely on a standard VAR approach to study the responses of hedge fund return moments (i.e., beta co-skewness and co-kurtosis) to macroeconomic and financial shocks. They innovate by introducing macroeconomic uncertainty in their study (Beaudry et al. 2001). They find that the beta is, among return moments, the most asymmetric to the business cycle and that hedge funds increase their beta during expansion and stabilize or decrease it during a recession.

The findings regarding the asymmetric responses of hedge fund strategies to macroeconomic and financial shocks are fragmentary.^11^ Some strategies, such as trend followers (CTA), futures, and equity market neutral, have received more attention than others. For instance, according to Criton and Scaillet (2011), the equity market neutral strategy has difficulty remaining neutral when market liquidity and volatility jump. Such a strategy is not neutral (Patton 2009). In contrast to many other strategies, the futures strategy seems to be a good market timer. Indeed, using the method proposed by Treynor and Mazuy (1966), Asness et al. (2001) find that the response of the returns delivered by this strategy is convex with respect to the market risk premium. These findings are important because investors such as pension funds are in search of returns and diversification in the current era of very low bond yields. Since hedge funds seem to be an interesting outlet in the context of persistent low interest rates, it is crucial to study whether hedge fund strategies may offer good diversification benefits to investors, especially in times of turmoil. For instance, if the responses of hedge fund strategies to shocks (i.e., macroeconomic, volatility, and illiquidity shocks) are too homogeneous during crises, we may conclude that hedge funds cannot provide substantial diversification benefits to institutional investors. Heterogeneous behavior from hedge fund strategies—especially during crises—is desirable from the point of view of portfolio diversification and thus financial stability (Beaudry et al. 2001; Wagner 2010). Moreover, since most hedge fund strategies maintain long and short positions simultaneously in different proportions, it is also important to study the impact of these opposite positions on their relative risk-taking. Given the lack of empirical results regarding the responses of hedge fund strategies to shocks, this study seeks to highlight the differential behavior of hedge fund strategies in terms of systematic risk (beta) during the business cycle, especially during a recession, when positive payoffs and portfolio diversification are needed the most. In this context, we innovate by subjecting hedge funds to extreme risk scenarios in “Appendix 1” using the TVAR (Balke 2000). This procedure makes it possible to subject hedge fund strategies to two kinds of shocks—standard shocks and strong shocks—under two regimes: recession (crisis) and expansion. The transition from one regime to another is determined by an optimal procedure, as explained in “Appendix 1”.

## Data and stylized facts

### Data

Data on quarterly hedge fund returns are drawn from a database managed by Greenwich Alternative Investment (GAI),^12^ who manage one of the oldest hedge fund databases, containing more than 13,500 records of individual hedge funds. The returns provided by the database are net fees. Our database runs from the first quarter of 1988 to the second quarter of 2016, for a total of 116 observations, a reasonable number on which to perform our VAR analyses. We rely on quarterly data for two main reasons. First, the GAI quarterly database spans a longer period than its monthly counterpart does.^13^ Second, because our empirical methodology is based on VARs, using quarterly data leads to less noise in the computation of the IRFs than would be generated by the use of monthly observations. Indeed, VARs are usually estimated using quarterly time series in the economic literature. In addition to the weighted composite index, our database includes 14 strategies, which are described in Table 1. Data for U.S. macroeconomic and financial variables are taken from the FRED database managed by the Federal Reserve Bank of St. Louis.

**Table 1:** Description of hedge fund strategies

Strategy	Description
convertibles	They take a long position in convertibles and short simultaneously the stock of companies having issued these convertibles in order to hedge a portion of the equity risk
Distressed securities	The managers buy equity and debt at deep discounts issued by firms facing bankruptcy. They may be involved in a capital structure game, going long and short on different securities of the same issuer such as long on debt and short on equity. This investment style may also be pursued in the derivatives market with structured products like CDS and other credit derivatives
Equity market neutral	The managers aim at obtaining returns with low or no correlation with equity and bond markets. They exploit the pricing inefficiencies between related equity securities
Event-driven	The managers follow a multistrategy event-driven approach
Fixed income	The managers follow a variety of fixed income strategies like exploiting relative mispricing between related sets of fixed income securities. They invest in MBS, CDO, CLO and other structured products. One of their main strategies is to go long on high yield or speculative bonds and short on bonds of higher credit ratings. The return spread between these two bond categories is a source of income for this strategy. However, this operation may provide substantial losses to the fixed income strategy when markets become illiquid or volatile
Futures	The manager utilizes futures contracts to implement directional positions in global equity, interest rate, currency and commodity markets. He takes long and short positions in these markets so its portfolio beta tends to revert to 0. He resorts to leveraged positions to increase his return. Similarly to short-sellers, this strategy is part of the long volatility category
Growth	The managers invest in companies experiencing strong growth in earnings per share
Long-short credit	They take long and short positions in credit in spite of the unavailability of bonds. They invest in high-yield bonds, CDS and CDO, among others
Macro	These funds have a particular interest in macroeconomic variables. They take positions according to their forecasts of these variables. Managers rely on quantitative models to implement their strategies
Mergers	These funds may purchase the stock of a company being acquired and simultaneously sell the stock of his bidder. They hope to profit from the spread between the current price of the acquired company and its final price
Multi-Strategy index	The manager utilizes investment strategies from more than one of the four broad strategy group indices
Opportunistic	The managers’ investment approach changes over time to better take advantage of current market conditions and investment opportunities
Short sellers	Managers take advantage of declining stocks. Short-selling consists in selling a borrowed stock in the hope of buying it at a lower price in the short-run. Managers’ positions may be highly leveraged. Like futures, this strategy is classified in the long volatility category
Value index	Managers invest in securities which are perceived undervalued with respect to their “fundamentals”. This strategy tends to resort to leverage to increase returns

*Sources:* Greenwich Global Hedge Fund Index Construction Methodology, Greenwich Alternative Investment (2015); Saunders et al (2014)

Many biases must be addressed when using hedge fund data, the major one being the survivorship bias (created when a database reports information on operating funds only; Capocci and Hübner 2004; Fung and Hsieh 2004; Patton et al. 2015). This bias is accounted for in the GAI database, as index returns include defunct funds since 1994.^14^ The GAI database also tackles self-selection bias and early reporting bias (Capocci and Hübner 2004; Fung and Hsieh 2004). Additional problems related to hedge fund returns are due to illiquidity and the practice of return smoothing (Asness et al. 2001; Amihud 2002, 2019; Pástor and Stambaugh 2003, 2019; Getmansky et al. 2004). These problems may lead to an underestimation of risk in the hedge fund industry. However, according to Asness et al. (2001), some biases—such as that due to the practice of return smoothing—should be less serious for quarterly data (the frequency used in this study) than for monthly data (the most popular frequency in hedge fund studies). This is another reason why we rely on quarterly data,^15^ in addition to the spanning argument advanced by Shiller and Perron (1985).

### Building time-varying betas

Most studies rely on the Kalman filter to compute time-varying risk measures, which then become state (unobserved) variables that obey a standard random walk model with drift or to a more complex model including macroeconomic and financial variables (Holmes and Faff 2008; Thomson and van Vuuren 2018; Lambert and Platania 2020).^16^ A more natural way to compute a time-varying beta is to rely on the Multivariate Generalized Autoregressive Conditional Heteroskedasticity process (MGARCH: Bollerslev et al. 1988; DCC-MGARCH: Engle and Colacito 2006; Engle 2016), which is specifically designed to build the conditional (time-varying) covariances that are the components of the time-varying beta measure.

The conditional beta ($\beta_{it}$) of strategy *i* is equal to

2 $$\beta_{it} = \frac{{{\text{cov}}_{t} \left( {R_{i} ,R_{m} } \right)}}{{{\text{var}}_{t} \left( {R_{m} } \right)}}$$

where the subscript *t* indicates that the beta measure is time-varying; cov_t_(*R*_*i*_*, R*_*m*_) is the conditional covariance between the return on the portfolio of strategy *i* (*R*_*i*_) and the market return (*R*_*m*_); and var_t_(*R*_*m*_) is the conditional variance of the market return.

To implement the MGARCH procedure, we compute the covariance appearing in Eq. (2) using the following simple system:

$$R_{it} = \gamma_{i} + \varepsilon_{it}$$

3 $$R_{jt} = \gamma_{j} + \varepsilon_{jt}$$

where *R*_*it*_ is the return of strategy *i* and *R*_*jt*_ is the market return; $\gamma_{i}$ and $\gamma_{j}$ are constants; and $\varepsilon_{it}$ and $\varepsilon_{jt}$ are innovations. The conditional covariance between *R*_*it*_ and *R*_*jt*_ is written as follows (Mills 1993):

4 $$cov_{ijt} = h_{ijt} = c_{ij} + a_{ij} \varepsilon_{it - 1} \varepsilon_{jt - 1} + b_{ij} h_{ijt - 1}$$

where *h*_*ijt*_ is an element of the conditional covariance matrix located in the ith and jth columns. Thus, we apply MGARCH to System (3) to compute *cov*_*t*_*(R*_*i*_*,R*_*m*_*)* in Eq. (2). The estimated conditional covariance matrix also provides an estimator of the conditional variance of the market return, which is required to compute the beta of a strategy (Eq. (2)). Note that we do not impose any particular structure on the covariances in System (3) because we aim to examine how hedge fund betas respond to macroeconomic and financial variables. Finally, we rely on the BEKK algorithm (Engle and Kroner 1995; Engle and Colacito 2006) to estimate the MGARCH because it is a parsimonious approach in terms of the number of parameters in the estimation.

Since we deal with time-varying beta, it is instructive to recall that an increase in the beta of a portfolio amounts to an increase in its financial leverage and that, by contrast, a decrease in beta is associated with a concomitant drop in leverage (i.e., a deleveraging process). Indeed, the following relationship exists between the beta of the stock issued by firm *i* and its leverage^17^ (Copeland et al. 2005):

5 $$\beta_{iL} = \beta_{iU} \left[ {1 + \left( {1 - t_{i} } \right)L_{i} } \right]$$

where $\beta_{iL}$ is the levered beta of firm *i*; $\beta_{iU}$ is its unlevered beta; *t*_*i*_ is its taxation rate; and *L*_*i*_ is its leverage, defined as the ratio of debt to equity. Hence, the levered beta of a firm co-moves positively with its leverage; thus, $\frac{{\partial \beta_{iL} }}{{\partial L_{i} }} = 1 - t_{i}$. Analogously, when considering a portfolio like the one held by a hedge fund, its beta co-moves positively with the leverage of the portfolio. Equation (5) proves useful in interpreting our results. Moreover, this equation allows us to compare our results with the findings of researchers that approach market timing from the angle of leverage rather than beta. Ang et al. (2011) find that hedge funds exhibit a high degree of similarity in their leverage exposures, which largely depends on market volatility (VIX) and the phase of the business cycle. In their study, hedge fund leverage and VIX move in the opposite direction, particularly during the subprime crisis, while a wave of deleveraging is observed in their sample of hedge funds. This was not the case for other financial institutions, especially investment banks, which saw their leverage soar during the crisis. Our results are quite similar to those of Ang et al. (2011) if we substitute beta for leverage as a measure of risk.^18^ However, our findings are more nuanced because we rely on more dynamic econometric methods. As explained later, we find that some strategies—countercyclical ones—deliberately increase their beta (leverage) during crises to capture risk premia or spreads related to their activities. Moreover, several strategies have difficulty reducing their beta during bad times, particularly in the face of extreme shocks.

### Descriptive statistics on strategies’ time-varying betas

Table 2 provides the descriptive statistics of the time-varying betas for the hedge fund general index and strategies. As expected, the average beta of the general index, at 0.38, is relatively low and fluctuates in an interval ranging from 0.20 to 0.53 over our sample period. The strategies with the highest average betas (in absolute values) are short sellers (− 0.98), growth (0.84), value index (0.59), opportunity index (0.47), event-driven (0.44), and multistrategy (0.40). The standard deviations of these betas are also relatively high, and we expect their behavior to be quite responsive to business cycles and financial conditions. Conversely, the strategies with the lowest average betas are futures (0.004), macro (0.11), long-short credit (0.13), equity market neutral (0.14), and fixed income (0.16). Therefore, the returns of these strategies are explained less by CAPM.

**Table 2:** Descriptive statistics of the time-varying betas of hedge fund strategies

	Mean	Median	Max	Min	St-dev	Sharpe	ρ
General index	0.378	0.389	0.532	0.203	0.078	4.846	0.25***
Convertibles	0.246	0.236	0.560	0.009	0.115	2.139	0.57***
Distressed securities	0.376	0.397	0.579	0.099	0.113	3.327	0.47***
Equity market neutral	0.145	0.156	0.219	0.023	0.047	3.085	0.27***
Event driven	0.439	0.460	0.651	0.185	0.113	3.885	0.29***
Fixed income	0.158	0.129	0.413	−0.098	0.114	1.386	0.41***
Futures	0.004	−0.007	0.245	−0.137	0.078	0.051	0.01
Growth	0.844	0.890	1.143	0.418	0.187	4.513	0.19**
Long-short credit	0.129	0.118	0.314	−0.109	0.085	1.518	0.39***
Macro	0.110	0.112	0.190	0.023	0.035	3.143	0.04
Mergers	0.136	0.141	0.221	0.029	0.048	2.833	0.23***
Multistrategy	0.401	0.429	0.564	0.165	0.099	4.051	0.24***
Opportunity index	0.470	0.481	0.711	0.268	0.096	4.896	0.20**
Short-sellers	−0.976	−0.949	−0.264	−1.922	0.351	−2.781	0.11*
Value index	0.591	0.610	0.821	0.298	0.131	4.511	0.18***

The time-varying beta of strategy *i* is equal to: $\beta_{it} = \frac{{{\text{cov}}_{t} \left( {R_{i} ,R_{m} } \right)}}{{{\text{var}}_{t} \left( {R_{m} } \right)}}$, where *R*_*i*_ is the return of strategy *i* and *R*_*m*_ is the market return, as measured by the S&P500’s return. The covariance and variance measures are computed with the MGARCH algorithm (Bollerslev et al. 1988; Engle and Colacito 2006; Engle 2016). The ρ coefficient associated with a strategy is equal to the coefficient of autocorrelation of the first-order degree of its return. The significance levels of the p-values are 1% indicated by ***; 5%, **; and 10%, *.

### Stylized facts

Table 2 also provides the first-order autocorrelation coefficients of the returns of hedge fund strategies. Billio et al. (2012) argue that hedge funds display the highest autocorrelation coefficient among the U.S. financial institutions they study (i.e., banks, brokers/dealers, insurers, and hedge funds). The degree of liquidity of a portfolio is negatively related to this coefficient (Lo 2001; Getmansky et al. 2004; Hasanhodzic and Lo 2007). In other words, the higher the coefficient, the greater the exposure to illiquidity. Strategies with a high autocorrelation coefficient should also be the most involved in the practice of return smoothing, since the degree of smoothing is positively related to the level of portfolio illiquidity. We conjecture that the most “liquid” hedge fund strategies according to this measure (i.e., futures, macro, short sellers, value index, and growth) are the most likely to succeed in rebalancing their portfolios following macroeconomic and financial shocks. By contrast, the strategies that are less liquid according to the autocorrelation measure (i.e., convertibles, distressed, fixed income, and long-short credit) are the most prone to have difficulty tackling shocks. Note that this category of illiquid strategies is involved in risky bonds such as mortgage-backed securities or high-yield bonds, which may be quite illiquid during bad times. In "Empirical results" section, we relate the degree of autocorrelation of strategies’ returns to their loadings to illiquidity measures—that is, the non-tradable and tradable illiquidity measures of Pástor and Stambaugh (2003) and the illiquidity ratio developed by Amihud (2002).

We obtain a first grasp of beta cyclicality in the hedge fund industry by using the following regression borrowed from Stiroh (2004)^19^:

6 $$beta_{it} = \alpha + \sum\limits_{\tau = 1}^{n} {\varphi_{t - \tau } } beta_{it - \tau } + \sum\limits_{\tau = 0}^{m} {\delta_{t - \tau } } d\ln GDP_{t - \tau } + \varepsilon_{it}$$

where *beta*_*it*_ is the conditional beta of a strategy at time *t,* and *dlnGDP* is *GDP* growth.^20^ Because the *dlnGDP* variable is autocorrelated, we rely on Almon’s (1965) polynomials to estimate the coefficients on the lags of it. This method can be easily implemented as follows. We assume the general finite-distributed lag model

7 $$Y_{t} = \alpha + \sum\limits_{i = 0}^{k} {\beta_{i} } X_{t - i} + \varepsilon_{t}$$

If we directly estimate (7) via OLS, the coefficients $\beta_{i}$ will have a low degree of significance because the lagged values of *X*_*t*_ are usually autocorrelated, resulting in a high degree of collinearity. The OLS will then be inefficient. To address this problem, Almon (1965) proposed approximating $\beta_{i}$ by a polynomial function of lag (*i*):

8 $$\beta_{i} = a_{0} + a_{1} i + a_{2} i^{2} + .... + a_{m} i^{m} \quad m < k$$

This procedure may be justified by Weierstrass’s theorem, which asserts that a function that is continuous in a closed interval can be approximated over the whole interval by a polynomial (Johnston 1972, p. 294). Substituting (8) into (7) yields

9 $$Y_{t} = \alpha + \sum\limits_{i = 0}^{k} {a_{i} } Z_{it} + \varepsilon_{t}$$

where *Z*_*it*_ is a polynomial expression for *X*_*it*_*.* Eq. (9) is estimated using the OLS. The coefficients $\beta_{i}$ can be retrieved using Eq. (8). For instance, for a second-degree polynomial, we have

10 $$\hat{\beta }_{i} = \hat{a}_{0} + i\hat{a}_{1} + i^{2} \hat{a}_{2} \quad i = 0,...,k$$

In Table 3, the sum of the lags (including the time *t* loading) of GDP growth is equal to 0.331 for the beta of the general index, suggesting that the beta of the representative hedge fund is procyclical, in the sense that it increases in expansion and decreases in recession. Most of the beta’s response to GDP growth is observed at time *t* (0.762), and it progressively dies out thereafter. For strategies displaying procyclical beta, we also observe most of the reactions of their beta at time *t*. Thus, this reaction is quick and not persistent. Not surprisingly, the beta of the growth strategy is the most procyclical, with a sum of lags equal to 0.658. At time *t*, the coefficient of GDP growth, at 1.921, is high. The other strategies whose beta is quite procyclical (i.e., which display a positive sum of lags) are event-driven, multistrategy, value index, and macro. These strategies can be classified into the directional group. Surprisingly, the beta of the equity market neutral strategy shows procyclicality: Its sum of lags is equal to 0.195, with a coefficient of 0.481 at time *t*, significant at the 1% level.

**Table 3:** Link between the betas of hedge fund strategies and GDP growth

	GDP growth								Adjusted
	t	t − 1	t − 2	t − 3	t − 4	t − 5	t-6	Sum of lags	R^2^
General index	0.762	0.168	− 0.212	− 0.379	− 0.333	− 0.074	0.399	0.331	0.93
	*2.51*	*1.14*	− *1.46*	− *2.22*	− *2.19*	− *0.52*	*1.44*		
Convertibles	0.492	− 0.311	− 0.584	− 0.328	0.458			− 0.273	0.95
	*1.06*	− *1.24*	− *1.82*	− *1.35*	*1.05*				
Distressed securities	0.399	− 0.222	− 0.591	− 0.708	− 0.573	− 0.187	0.451	− 1.431	0.96
	*1.23*	− *1.46*	− *3.73*	− *3.78*	− *3.47*	− *1.15*	*1.38*		
Equity market neutral	0.481	− 0.018	− 0.239	− 0.182	0.153			0.195	0.96
	*3.01*	− *0.19*	− *2.14*	− *2.05*	*0.94*				
Event driven	1.003	0.148	− 0.379	− 0.579	− 0.451	0.005	0.789	0.535	0.95
	*2.60*	*0.77*	− *2.01*	− *2.64*	− *2.27*	*0.03*	*2.25*		
Fixed income	0.010	− 0.613	− 0.717	− 0.302	0.633			− 0.989	0.96
	*0.03*	− *3.11*	− *2.76*	− *1.48*	*1.67*				
Futures	0.195	− 0.053	− 0.203	− 0.256	− 0.212	− 0.071	0.167	− 0.434	0.96
	*0.93*	− *0.52*	− *1.91*	− *2.07*	− *1.98*	− *0.70*	*0.80*		
Growth	1.921	0.175	− 0.720	− 0.763	0.045			0.658	0.95
	*2.73*	*0.43*	− *1.45*	− *2.00*	*0.06*				
Long-short credit	0.228	− 0.118	− 0.304	− 0.330	− 0.196	0.098	0.552	− 0.070	0.95
	*0.88*	− *0.95*	− *2.39*	− *2.22*	− *1.54*	*0.82*	*2.20*		
Macro	0.329	0.089	− 0.061	− 0.121	− 0.091	0.030	0.240	0.414	0.95
	*2.89*	*1.51*	− *1.05*	− *1.80*	− *1.49*	*0.51*	*2.30*		
Mergers	0.276	0.025	− 0.124	− 0.170	− 0.113	0.047	0.309	0.251	0.96
	*1.51*	*0.26*	− *1.40*	− *1.70*	− *1.22*	*0.51*	*1.89*		
Multistrategy	0.778	0.094	− 0.321	− 0.467	− 0.343	0.049	0.710	0.500	0.95
	*2.28*	*0.56*	− *1.92*	− *2.38*	− *1.95*	*0.30*	*2.29*		
Opportunity index	0.735	0.049	− 0.377	− 0.545	− 0.454	− 0.104	0.505	− 0.191	0.92
	*1.90*	*0.27*	− *2.03*	− *2.48*	− *2.35*	− *0.58*	*1.40*		
Short-sellers	− 0.919	0.110	0.793	1.131	1.124	0.772	0.074	3.012	0.95
	− *1.03*	*0.23*	*2.15*	*2.49*	*2.24*	*1.70*	*0.20*		
Value index	1.324	0.269	− 0.404	− 0.694	− 0.602	− 0.126	0.732	0.498	0.95
	*2.88*	*1.18*	− *1.83*	− *2.70*	− *2.61*	− *0.57*	*1.73*		

The coefficients are obtained by regressing returns on an Almon (1965) polynomial defined on GDP growth as measured with $d\ln GDP \times 100$. The number of lags can differ from one strategy to the next. Except for short-sellers, a positive (negative) sign for the sum of lags indicates a procyclical (contracyclical) strategy’s beta. The magnitude of the sum of lags indicates the degree of beta’s cyclicality. t-statistics are in italics.

By contrast, the betas of some strategies are countercyclical in the sense that they tend to decrease in expansion and increase in recession. In this respect, short sellers are the most countercyclical strategies. The sum of lags for GDP growth at 3.012 is high. Since the beta of short sellers is negative, an increase in GDP growth results in an increase in its beta, which corresponds to a decrease in absolute value. This finding suggests that short sellers take less systematic risk in expansion and take more in recession, which corresponds to a countercyclical beta. This behavior is associated with contrarian operations. Note that the reaction of short sellers’ beta to GDP growth is delayed. Significant coefficients are observed only after two quarters. This is the case for all other countercyclical strategies that respond to GDP growth with a delay, in contrast to procyclical ones. At time *t*, the loading of their beta to GDP growth is positive but not significant. As shown in the empirical section, countercyclical strategies seek to capture risk premia associated with non-traditional sources of risk during recessions (crises), and this behavior is associated with an increase in the systematic risk they bear—that is, their beta. However, the reaction of their beta may be delayed because the turning points of the business cycle are difficult to predict. Furthermore, two strategies quite involved in bond markets—distressed and fixed income—also display countercyclical behavior, with their sum of lags being negative.^21^ Their beta may take negative values, as indicated by the column of minima in Table 2, which is also an indication of a countercyclical pattern. This behavior is partly due to the fact that bond prices are classified as countercyclical indicators. In the empirical section, we also show that this countercyclical behavior is associated with the market timing activities of the corresponding strategies. In this respect, note that the futures strategy also displays countercyclicality, the sum of lags related to GDP growth being equal to − 0.434 for this strategy, whose beta may also take negative values (see Table 2). According to Stafylas et al. (2018), this strategy follows a countercyclical policy during recessions by investing in commodities whose demand is inelastic to prices, especially food, energy, and industrial metals. Hence, these commodities are associated with defensive or countercyclical industries.

The remaining strategies are more difficult to classify. Their beta does not display a clear response to GDP growth. This is especially true for the long-short credit strategy, for which the sum of lags is slightly negative (− 0.07), and whose beta may also take negative values (see Table 2). It would thus appear to behave more like a countercyclical strategy than a procyclical one. The sum of lags is higher in absolute value for the convertible strategy, being equal to − 0.273, but the coefficients associated with the lags of GDP growth are not very significant, and the minimum of its beta is close to 0. Indeed, this strategy holds shares that have two dimensions, a bond and a stock, as embedded in the call option incorporated in the convertible bond. The stock dimension tends to make this strategy procyclical, while the bond dimension tends to make it countercyclical. The significance of the coefficients associated with the mergers strategy is also quite low, although the sum of lags is positive for its coefficients, being equal to 0.251. Thus, it should be placed in the procyclical category. Finally, the sum of lags is negative for the opportunity index strategy, even if its beta responds positively and significantly to GDP growth at time *t*. At 0.735, the corresponding coefficient is quite high. Overall, the response of the time-varying beta of hedge fund strategies to GDP growth may be quite complex, as it is associated with the idiosyncrasy of their market timing activities. We shed more light on this issue in the section below that delivers our empirical results.

There are other ways to classify hedge fund strategies. We can distinguish short volatility strategies from long volatility strategies (Mitchell and Pulvino 2012; Page and Panariello 2018; Lambert and Platania 2020). Page and Panariello (2018) claim that most hedge funds are in the short volatility category; thus, they are involved in short-volatility trades. They make money when market volatility decreases, like a long-call butterfly spread. However, there are at least two notable exceptions: short sellers and futures. In our sample, the correlation between their returns and VIX is approximately 0.25 for both, significant at the 5% level. They behave as straddles, making money when market volatility increases. Thus, these strategies may offer positive payoffs to investors when market volatility jumps.

Combining strategies may offer beneficial diversification potential for investors, especially during crises, when strategies are classified as procyclical or countercyclical. Indeed, there is a good balance between the two categories. This is less true if we classify hedge fund strategies into long and short volatility types, since most hedge fund strategies are in the short volatility category. If VIX shocks are more important than GDP growth shocks in explaining the behavior of strategies’ betas, the diversification benefits associated with the pooling of different strategies in a single portfolio are reduced.

## Empirical results

In this section, we rely on Jordan’s (2004, 2005, 2009) LP method to assess the nonlinearities associated with the behavior of hedge fund strategies’ beta during the subprime crisis (from the third quarter of 2007 to the fourth quarter of 2009). The results are then compared to the benchmark standard linear VAR, which displays the “average” beta behavior over the business cycle. “Appendix 1” shows the results of using another approach to studying the market timing activities of hedge fund strategies with respect to their beta, the TVAR as developed by Balke (2000). This method melts all crisis or recession periods to obtain an optimal transition from the low to the high regime.

### Description of LP method

In the LP method, the coefficients are not computed recursively, as in the standard linear VAR, but are based on the following expression of an optimal forecast (Jordà 2004, 2005; Misina and Tessier 2008; Tessier 2015), which, incidentally, corresponds to the general definition of an impulse response (Ghysels and Marcellino 2018; Koop et al. 1996; Franses and van Dijk 2000):

11 $$IR\left( {t,s,{\mathbf{d}}_{{\mathbf{i}}} } \right) = E\left( {{\mathbf{y}}_{{{\mathbf{t + s}}}} \left| {{\mathbf{v}}_{{\mathbf{i}}} = {\mathbf{d}}_{{\mathbf{i}}} } \right.;{\mathbf{X}}_{{\mathbf{t}}} } \right) - E\left( {{\mathbf{y}}_{{{\mathbf{t + s}}}} \left| {{\mathbf{v}}_{{\mathbf{i}}} = {\mathbf{0}}} \right.;{\mathbf{X}}_{{\mathbf{t}}} } \right)\quad s = 0,1,2,...,h$$

where *IR(.)* is the impulse response at time *t* + *s*, the length of the horizon being h; *E(.)* denotes the best mean-squared error predictor; **y**_**t**_ is the $\left( {k \times 1} \right)$ vector of endogenous variables,${\mathbf{X}}_{{\mathbf{t}}} = \left( {y_{t - 1} ,y_{t - 2} ,...} \right)^{\prime}$; ${\mathbf{0}}$ is of dimension $\left( {k \times 1} \right)$; **v**_**i**_ is the $\left( {k \times 1} \right)$ vector of reduced-form disturbances; and **d**_**i**_ is the vector of the experimental shocks. An impulse response is thus the difference between two forecasts: (i) the forecast of **y**_**t+s**_ when the instantaneous structural shocks are equal to **d**_**i**_ and (ii) the forecast of **y**_**t+s**_ when the structural shocks are null.

To compute the IRFs associated with the LP method, we must reserve the last *s* + *1* observations of the sample as dependent variables of the local projection (i.e., from **y**_**t**_ to **y**_**t+s**_). We regress these vectors sequentially on the same set of lagged variables; thus,$\left( {{\mathbf{y}}_{{{\mathbf{t - }}1{\mathbf{,}}}} {\mathbf{y}}_{{{\mathbf{t - 2}}}} {\mathbf{,}}...{\mathbf{,y}}_{{{\mathbf{t - p}}}} } \right)^{\prime}$. In other words, we project ${\mathbf{y}}_{{{\mathbf{t + s}}}}$ onto the linear space generated by $\left( {{\mathbf{y}}_{{{\mathbf{t - 1}}}} {\mathbf{,y}}_{{{\mathbf{t - 2}}}} {\mathbf{,}}...{\mathbf{,y}}_{{{\mathbf{t - p}}}} } \right)^{\prime}$ (Jordà 2004, 2005; Kilian and Kim 2011):

12 $${\mathbf{y}}_{{{\mathbf{t + }}s}} {\mathbf{ = }}\alpha^{s} + {\mathbf{F}}_{{\mathbf{1}}}^{s + 1} {\mathbf{y}}_{{{\mathbf{t - 1}}}} {\mathbf{ + F}}_{{\mathbf{2}}}^{s + 1} {\mathbf{y}}_{{{\mathbf{t - 2}}}} {\mathbf{ + }}...{\mathbf{ + F}}_{{\mathbf{p}}}^{s + 1} {\mathbf{y}}_{{{\mathbf{t - p}}}} {\mathbf{ + \mu }}_{t + s}^{s} \quad s = 0,...,h$$

where $\alpha^{s}$ is a $\left( {k \times 1} \right)$ vector of constants, and ${\mathbf{F}}_{i}^{s + 1}$ are matrices of coefficients for each lag *i* and horizon *s* + *1.*

There are two procedures for computing multistep forecasts (Tessier 2015): (i) dynamic simulation, as in the standard linear VAR; and (ii) specifying direct forecasting models that are reestimated for each horizon. According to Jordà (2004, 2005), the latter method is more robust to misspecifications of the data generating process (DGP), thereby avoiding the accumulation of misspecification errors through the nonlinear calculation of the conventional VAR technique as the forecast horizon lengthens. Moreover, Montiel Olea and Plagborg-Moller (2021) assert that the LP method is more robust than standard autoregressive inference (i.e., plain vanilla linear VAR), whose validity is known to depend sensitively on the persistence of the data and the length of the horizon, which is not the case for the LP procedure.

The estimated impulse responses ($\widehat{{{\mathbf{IR}}}}$) associated with Eq. (12) can be easily computed as

13 $$\widehat{{{\mathbf{IR}}}}\left( {t,s,{\mathbf{d}}_{{\mathbf{i}}} } \right) = \hat{F}_{{\mathbf{1}}}^{{\mathbf{s}}} {\mathbf{d}}_{{\mathbf{i}}} \quad s = 0,1,2,...,h$$

with the normalization ${\mathbf{F}}_{{\mathbf{1}}}^{{\mathbf{0}}} {\mathbf{ = I}}$. Only the estimated coefficients of the first lag in Eq. (12) enters the impulse response equations.

According to Jordà (2004, 2005), the impulse responses in the LP method are computed by a sequence of projections of the endogenous variables shifted forward into their own lags. The estimated coefficients of the LP model are time-varying because they are conditional on the value of *h*, the horizon of the LP. These projections are thus “local’ for each forecast horizon. They are not computed recursively, as in the classical linear VAR model.

The confidence interval (CI) of the IRFs computed for nonlinear local projections is easily obtained. The $\left( {1 - \alpha } \right)\%$ confidence interval of IRF is equal to

14 $$CI = \widehat{IR} \pm \left[ {z_{{1 - {\alpha \mathord{\left/ {\vphantom {\alpha 2}} \right. \kern-\nulldelimiterspace} 2}}} \times \left( {{\mathbf{d}}_{{\mathbf{i}}}^{{\mathbf{^{\prime}}}} {\hat{\mathbf{\Sigma }}}_{{\mathbf{C}}} {\mathbf{d}}_{{\mathbf{i}}} } \right)} \right]$$

where $${{\varvec{\Sigma}}}_{{\mathbf{C}}}$$ is the HAC variance–covariance matrix of the coefficients $$\hat{F}_{i}^{s}$$ in Eq. (12), and $$z_{{1 - {\alpha \mathord{\left/ {\vphantom {\alpha 2}} \right. \kern-\nulldelimiterspace} 2}}}$$ denotes the $$\left( {1 - {\raise0.7ex\hbox{$\alpha $} \!\mathord{\left/ {\vphantom {\alpha 2}}\right.\kern-\nulldelimiterspace} \!\lower0.7ex\hbox{$2$}}} \right)$$ quantile of the N(0,1) distribution.

### The empirical model

To implement the local projection of the strategies’ beta, we rely on the following parsimonious^22^ macroeconomic model, which is indebted to the empirical APT model of Chen et al. (1986) and to the multifactorial model proposed by Gregoriou (2004) for studying the market timing of hedge funds:

15 $$\beta_{it} = \lambda_{0} + \lambda_{1} d{\text{lnGDP}}_{t} + \lambda_{2} credit\_spread_{t} + \lambda_{3} term\_spread_{t} + \lambda_{4} VIX_{t} + \varsigma_{it}$$

where $\beta_{it}$ is the time-varying beta of strategy *i*, *dlnGDP*_*t*_ is the U.S. GDP growth rate, *credit_spread*_*t*_ is the spread between BBB and AAA corporate bond yields, *term_spread*_*t*_ is the spread between the 10-year interest rate and the three-month Treasury bill rate, and *VIX*_*t*_ measures the volatility of the U.S. stock market. Note that the choice of the term spread, credit spread, and GDP growth rate is supported by many hedge funds studies (Kat and Miffre 2002; Amenc et al. 2003; Brealy and Kaplanis 2010; Bali et al. 2014; Lambert and Platania 2016, 2020). Furthermore, Agarwal et al. (2017) and Lambert and Platania (2016, 2020) find that the implied volatility of the U.S. stock market (VIX) is an important driver of hedge fund returns and hedge fund exposure to the stock market and to the Fama and French (1992, 1993, 2015) factors. Finally, following Gregoriou (2004), the model we use is justified by the fact that hedge funds trade options (*VIX*) and have significant exposure to credit risk (*credit_spread*) and term spread risk (*term_spread*). Taking short and long positions on securities simultaneously to capture yield spreads is indeed an important source of income for hedge funds involved in arbitrage. In addition to these variables, we include GDP growth to study the procyclicality of the risk borne by hedge fund strategies.

After many experiments on different orderings involving the classical information criteria to select a VAR model (i.e., the AIC, AIC_c_,^23^ and SIC), we order the variables of our setting in the following sequence, from the most exogenous to the most endogenous:

$$d\ln GDP \to term\;spread \to credit\;spread \to beta \to VIX$$

GDP growth is therefore considered the most exogenous variable, while *VIX*, which is very sensitive to economic news, is viewed as the most endogenous. At the impact, the beta reacts to GDP growth, the term spread, and the credit spread, but not to the *VIX*. Note that this ordering is empirical because there is no comprehensive theoretical framework on the co-movements between the variables included in our VAR model.

### Results

#### *Negative GDP growth shocks*^24^

**Fig. 1:**
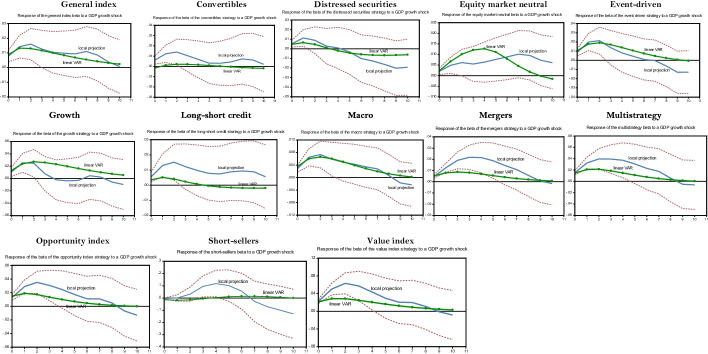
Local projection of a GDP growth shock on hedge fund strategies’ betas over the subprime crisis. *Notes:* The impulse response functions corresponding to the linear LP—i.e., the beta forecast following external shocks—are built using the Jordà’s (2004, 2005, 2009) algorithm provided by Eren Ocakverdi, consultant at Yapy Kredi. The local projection of a beta strategy is performed over the subprime crisis (2007Q3–2009Q4). We also provide the linear VAR estimated over the whole sample period selected as benchmark. The confidence interval of the LP impulses, computed at the threshold of 95%, is in dotted lines. As explained in the paper, our model comprises five endogenous shocks, placed in the following order to perform the Cholesky factorization (Sims 1980) required to build structural shocks: GDP growth, term spread, credit spread, beta (own shock) and VIX. As usual, these variables are ordered by increasing level of endogeneity. This order relies on the conventional information criteria: Akaike and Schwartz criteria. Only strategies whose impulse response functions are significant at a level of 10% or less are reported in this table. Note that the IRFs are computed for positive GDP shocks but the corresponding IRFs associated with negative shocks are the mirror images of the former since, for both methods, the IRFs being computed with linear techniques. The fixed income and futures strategies are excluded since their IRFs are not enough significant

For our simulations during the subprime crisis, based on the usual information criteria (i.e., the AIC, AIC_c_, and SIC), we compute the IRFs of our models with three lagged values for our vector of explanatory variables. Figure 1 provides the IRFs of the LP of the beta associated with a GDP growth shock and the corresponding IRFs computed using a standard linear VAR as a benchmark.

The response of the general index to a GDP shock is moderate over the subprime crisis, as computed using the LP method. We do not detect the presence of any significant asymmetries because the plots associated with LP and the benchmark VAR are similar. Hence, the decrease in the beta of the general index during the crisis after a negative GDP shock is close to its average response over the entire sample period. This similarity between the IRFs computed with LP and the linear VAR is shared by other strategies, such as macro, distressed, event-driven, and growth. For these two last strategies, the IRFs computed with LP are lower than are those built with the linear VAR, suggesting that these strategies had difficulty in reducing their beta during the crisis. The case of the distressed securities strategy is particularly interesting because, after the occurrence of a negative GDP growth shock, its IRF turns upward after some quarters to become positive. This strategy thus seizes profitable opportunities during the subprime crisis due to the large number of firms facing difficulties, but its reaction was delayed consistent with our previous analysis on the cyclicality of hedge fund strategies.

However, we observe significant asymmetries for many strategies, such as short sellers, value index, long-short credit, mergers, opportunity index, convertibles, and multistrategy. Indeed, excluding short-sellers, the negative response of the beta to a negative GDP shock is significantly higher with LP. This means that these strategies focused on channeling systematic risk during the subprime crisis. The convertible strategy deserves more attention. During the subprime crisis, the decline in its beta after a negative GDP shock is related to the two dimensions of a convertible bond—(i) a bond and (ii) a stock—as embedded in its call option. Bond dimensions dominate during financial crises. A negative GDP shock tends to reduce the beta of the convertible strategy because the call option incorporated in the convertible bond becomes out-of-the-money. Since the beta of a bond is lower than the beta of a stock, the beta of the convertible strategy decreases after a negative GDP growth shock.

Strategies linked to market events (i.e., mergers, opportunity index, and multistrategy) seem to have also performed more hedging transactions in order to better capture the risk premia related to their specialized activities after adverse GDP shocks. By contrast, the short-sellers’ IRF computed with the benchmark linear VAR indicates a marginal increase in beta (in absolute value) after a negative GDP shock. However, this increase is much higher when LP is used, as the IRF peaks at 0.1. Given their contrarian operations, short-sellers therefore benefit from turmoil since they rely on financial leverage to boost their beta (i.e., Eq. (5)) during these periods.

#### Positive VIX shocks

**Fig. 2:**
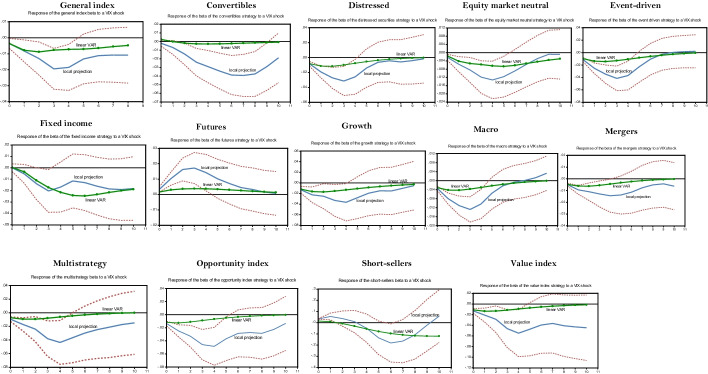
Local projection of a VIX shock on hedge fund strategies’ betas over the subprime crisis. *Notes:* The impulse response functions corresponding to the linear LP are built using the Jordà’s (2004, 2005, 2009) algorithm provided by Eren Ocakverdi, consultant at Yapy Kredi. The local projection of a beta strategy is performed over the subprime crisis (2007Q3–2009Q4). We also provide the linear VAR estimated over the whole sample period selected as benchmark. The confidence interval of the LP impulses, computed at the threshold of 95%, is in dotted lines. As explained in the paper, our model comprises five endogenous shocks, placed in the following order to perform the Cholesky factorization (Sims 1980) required to build structural shocks: GDP growth, term spread, credit spread, beta (own shock), and VIX. As usual, these variables are ordered by increasing level of endogeneity. This order relies on the conventional information criteria: Akaike and Schwartz criteria. The long-short credit strategy is excluded since its IRF is not enough significant

According to Black’s (1976) leverage effect, a positive VIX shock is associated with falling stock markets. The response of the betas of the general index and of most strategies is much greater to this financial shock than it is to a negative GDP growth shock (see Fig. 2). More importantly, for most strategies, asymmetries—as measured by the spread between the IRFs computed with LP and with the benchmark linear VAR—are much higher than in the case of an unfavorable GDP growth shock. Indeed, the negative response of the beta to positive VIX shocks is generally more important with LP than with linear VAR, suggesting that hedge funds reduced their systematic risk during the subprime crisis in a greater proportion than they did over the whole sample period after a VIX shock of similar amplitude. This indicates that the response of the beta to a given VIX shock is conditional on the degree of economic uncertainty, which was particularly high during the subprime crisis.

The response of the beta to a VIX shock, as measured with LP, is relatively high for procyclical strategies (i.e., value index, growth, multistrategy, opportunity index, and event-driven). This suggests that VIX plays a key role in hedge fund risk management. The value index case is particularly interesting. According to the linear VAR, the decrease in its beta is relatively low following a VIX shock, the IRF peaking at − 0.01. However, the peak of the IRF measured with LP is close to − 0.06 and is significant at the 5% level. Note that the value index strategy is quite liquid because its first-order autocorrelation coefficient, a proxy of illiquidity risk, is low (see Table 3). This facilitates the adjustment of beta following an adverse VIX shock. This is also the case for most other procyclical strategies. Incidentally, the response of the market-neutral strategy beta to a VIX shock is similar to that of procyclical strategies because its decrease is greater with LP than with linear VAR. Neutrality should mean that the beta of this strategy should be insensitive to the VIX; however, we find that this is not the case during the subprime crisis (Asness et al. 2001; Patton 2009; Criton and Scaillet 2011).

The beta of the convertible strategy tends to decrease after a VIX shock. In this respect, we note that this decrease was substantial during the subprime crisis when measured with LP since it peaked at − 0.04, while it was negligible when computed with the linear VAR, an obvious case of asymmetry. After a VIX shock, which tends to increase the value of the call embedded in the hybrid securities, the convertible strategy seems to quickly hedge this rising equity risk. Note that the convertible strategy usually hedges equity risk so that its payoffs are insensitive to this kind of risk. In other respects, the IRF of the beta of the distressed securities strategy estimated with LP decreases at the impact of a VIX shock during the subprime crisis, but it trends clearly upward after a delay of some quarters. This is due to the countercyclical dimension of this strategy, as explained in "Stylized facts"  section. As shown in this section, this countercyclicality is delayed, and it aims to capture the substantial spreads related to the rising number of distressed firms during crises. In other respects, the beta of the fixed income strategy decreased moderately when computed with the linear VAR, while the LP procedure suggests that this strategy had difficulties in dealing with VIX shocks during the subprime crisis.

Finally, the beta of the contrarian strategies (i.e., short sellers and futures) increased (in absolute value) during the subprime crisis after a positive VIX shock.^25^ For instance, when computed with LP, the IRF of short-sellers reached 0.2, a quite high level, while it increased less when computed by the standard linear VAR. The asymmetry is more pronounced for the futures strategy, for which the spread between the LP and linear VAR is even greater.

#### *Positive credit spread and term spread shocks*^26^

**Fig. 3:**
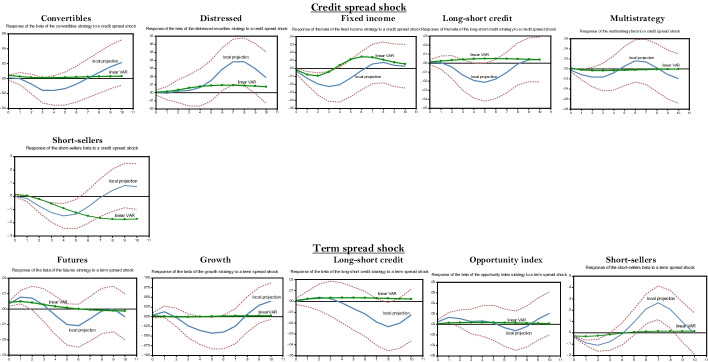
Local projection of a credit spread and term spread shocks on hedge fund strategies’ betas over the subprime crisis. *Notes:* The impulse response functions corresponding to the linear LP are built using the Jordà’s (2004, 2005, 2009) algorithm provided by Eren Ocakverdi, consultant at Yapy Kredi. The local projection of a beta strategy is performed over the subprime crisis (2007Q3–2009Q4). We also provide the linear VAR estimated over the whole sample period selected as benchmark. The confidence interval of the LP impulses, computed at the threshold of 95%, is in dotted lines. As explained in the paper, our model comprises five endogenous shocks, placed in the following order to perform the Cholesky factorization (Sims 1980) required to build structural shocks: GDP growth, term spread, credit spread, beta (own shock) and VIX. As usual, these variables are ordered by increasing level of endogeneity. This order relies on the conventional information criteria: Akaike and Schwartz criteria. Only strategies whose impulses response functions are significant at a level of 10% or less are reported in this table

The credit spread is a countercyclical indicator that tends to be high during a recession and low in expansion. A positive credit spread shock corresponds to a deterioration in corporate solvency. As expected, these kinds of shocks mostly impact strategies that are particularly involved in bond markets—especially convertibles, distressed securities, fixed income, long-short credit, and multistrategy (see Fig. 3). The distressed securities strategy seems to benefit from a credit spread shock since its beta increases afterward. Indeed, its main source of return is associated with the credit risk premium, which was especially high during the subprime crisis because credit risk embedded in mortgage-backed securities was its main driver. The IRF associated with the linear VAR suggests that this strategy also seeks to capture the credit risk premium during more normal times but less aggressively. The other ones reduce their systematic risk—that is, they deleverage—after this shock, which suggests that they are adversely affected by it. Asymmetries are at play because the reaction delivered by LP is usually much stronger than that associated with the linear VAR, especially for convertibles, distressed, long-short, and multistrategy, which display a weak reaction when the linear VAR is used but a more substantial one when the LP is used. Note that fixed income and long-short credit strategies may seek to capture the credit risk premium in more normal times. Indeed, their IRF, as computed with the linear VAR, trends upward in that case. Not surprisingly, short sellers increase their beta (absolute value) at the impact of a credit spread shock. This change is higher with LP than with the benchmark linear VAR.

A positive term spread shock is associated with an increase in market liquidity. Indeed, when financial markets become illiquid, investors buy short-term bonds and sell long-term bonds that are less liquid. These transactions increase the term spread. However, the term spread shock is also a countercyclical indicator that tends to be high in recession and low in expansion. Strategies are less sensitive to a term spread shock than to the others (see Fig. 3). At the impact of this shock, the beta of short-sellers increases significantly as measured with LP, while the corresponding IRF of the futures strategy shows a moderate increase. Thus, these strategies aim to capture the liquidity premium at the impact of the shock. However, after some quarters, the beta of the strategies reported in Fig. 3 (i.e., short sellers, futures, growth, long-short credit, and opportunity index) decreases significantly. This move may be related to the countercyclical dimension of the term spread indicator. Moreover, except for long-short credit, these strategies are among the most liquid according to their autocorrelation coefficients (see Table 2). Hence, increased market illiquidity, as measured by the term spread, seems to hamper their business lines. Indeed, these “liquid” strategies borrow heavily on the money markets (e.g., the repo market), and an increase in the term spread (i.e., a rise in market illiquidity) results in an increase in required margins by brokers, which reduces borrowings. Generally, credit frictions in financial markets lead to the presence of an external finance premium (Gertler and Karadi 2015). In this context, the interest rate charged to firm^27^*i* (*r*_*it*_) in the money market can be written as

16 $$r_{it} = rf_{t} + efp_{t}$$

where $rf_{t}$ is the risk-free rate and *efp*_*t*_ is the external finance premium. This premium increases when illiquidity, as measured by the term spread, trends upward, especially during crises, which results in an increase in borrowing costs for firm *i*. Moreover, according to the expectations hypothesis, an increase in the term spread signals a rise in future borrowing costs, which also induces hedge funds to lower their leverage (Ang et al. 2011). According to expectations theory, the m-period interest rate (*r*_*lt*_) is related to the one-period present and future rates *r*_*s*_ by the following equation (Gertler and Karadi 2015):

17 $${\text{Term spread }} = r_{lt} - r_{st} = E_{t} \left[ {\frac{1}{m}\sum\limits_{j = 0}^{m - 1} {r_{s,t + j} } } \right] + \phi_{tm} - r_{st}$$

where $\phi_{tm}$ is the term premium and *Et(.)* is the expectation of the mean of the actual and future short-term rates over the maturity of the long-term bond. According to Eq. (17), an increase in the term spread is associated with an increase in future borrowing costs, measured by the actual and future short-term rates. Finally, note that asymmetries are obvious regarding the response of the beta to a term spread shock. Indeed, for all sensitive strategies, this response is very low when measured using the benchmark linear VAR.

## Robustness check: response of hedge fund strategies to illiquidity shocks

Similar to volatility shocks, liquidity shocks are important during recessions or crises, while they are less so in normal times (Pástor and Stambaugh 2019). In this section, we investigate the extent to which hedge fund strategies manage liquidity shocks using indicators other than the term spread. This question is crucial because one of the main benefits of hedge funds for investors is their involvement in non-traditional types of risks, such as tail risk, credit risk, and liquidity risk (Hasanhodzic and Lo 2007). Before performing local projections on hedge fund conditional betas using illiquidity shocks, we regress hedge fund strategy returns and betas on illiquidity measures to obtain initial insights into the impact of illiquidity on the behavior of hedge funds. These experiments will help interpret the results obtained using the local projections. In “Appendix 1”, we rely on TVAR to gauge the response of hedge fund strategies’ betas to illiquidity shocks.

### Impact of illiquidity on hedge fund returns and conditional betas

We assess the impact of illiquidity on hedge fund returns and conditional betas with a pooled regression of hedge fund strategies using an augmented Fama and French (2015) five-factor model.^28^ This equation is as follows:

18 $$\begin{gathered} R_{it} - R_{ft} = \alpha + \theta_{1} (R_{mt} - R_{ft} ) + \theta_{2} SMB_{t} + \theta_{3} HML_{t} + \theta_{4} RMW_{t} + \theta_{5} CMA_{t} + \theta_{6} ILLIQ_{t} + ... \hfill \\ ... + \theta_{7} term\_spread_{t} + \theta_{8} dr10years + \theta_{9} credit\_spread_{t} + \varepsilon_{t} \hfill \\ \end{gathered}$$

where *R*_*it*_ is the return of strategy *i*; *R*_*ft*_ is the risk-free rate as measured by the three-month Treasury bill rate; *R*_*mt*_ is the return on the S&P500; *SMB*_*t*_ is the size factor; *HML*_*t*_ is the value factor; *RMW*_t_ is the profitability factor; *CMA*_*t*_ is the investment factor; *ILLIQ*_*t*_ is an illiquidity measure that takes two forms—(i) the Pástor and Stambaugh’s (2003) tradable illiquidity measure (*IML*_*t*_), a portfolio long in stocks with high liquidity betas and short in stocks with low liquidity betas, and (ii) the Amihud (2002) illiquidity ratio (*AMI*_*t*_), computed using the S&P500^29^; and *dr10years* is the first-difference in the 10-year Treasury rate. We also estimate Eq. (18), using the strategies’ time-varying (conditional) betas as dependent variables.^30^ This pooled regression employs seemingly unrelated regressions (SUR) to account for the interaction between the strategies’ innovations.^31^

We cope with the asymmetric impact of crises on the behavior of hedge funds by estimating Eq. (18) over two scenarios: (i) a scenario that distinguishes between periods of crises that occurred during our sampling period and periods outside these crises; and (ii) another scenario that separates the subprime crisis from the rest of the sample. To implement the first scenario, we multiply the explanatory variables in Eq. (18) by two dummy variables: (i) a dummy that takes a value of one for the crises observed over our sample period—that is, the Asian-LTCM-Russian crisis (third quarter of 1997 to fourth quarter of 1998), the tech-bubble crisis (fourth quarter of 2000 to the first quarter of 2003), and the subprime crisis (third quarter of 2007 to fourth quarter of 2009)—and 0 otherwise; and (ii) a dummy that takes a value of one for the period outside crisis and 0 otherwise. We proceed in the same way to devise the truncated variables of the second scenario, focusing on the subprime crisis.

#### Impact of IML on strategies’ returns

Table 4 provides the Pástor–Stambaugh (2003) loadings for strategy returns computed over our two crisis scenarios and over the whole sample period. The Pástor–Stambaugh (2003) factor is a measure of financial market illiquidity. The higher it is, the higher the degree of illiquidity. For a strategy, loading represents the exposure of its returns to this illiquidity factor. In the first scenario, illiquidity is mainly at play during crises, since there are few significant strategies’ loadings outside crises. We usually expect a positive sign for these loadings because illiquidity commands a risk premium (Amihud 2002; Pástor and Stambaugh 2003; Konstantopoulos 2016). However, this type of reasoning should be qualified. First, the illiquidity risk premium is concerned with the *expected* (forecasted) returns and not the *observed* returns used in regressions.^32^ During crises, increased illiquidity may, by generating uncertainty, result in a decrease in stock prices and thus in *observed* stock returns; we can then obtain a negative sign for the coefficients of the illiquidity measures even if expected returns embed an illiquidity premium (Acharya and Pedersen 2005; Konstantopoulos 2016). We call this effect the “uncertainty effect” related to illiquidity measures, which should mainly be at play during crises (Konstantopoulos 2016). While Amihud (2002) and Pástor and Stambaugh (2003) argue for a contemporaneous positive relationship between illiquidity and expected returns (i.e., a static relationship), the uncertainty effect establishes a dynamic link between illiquidity and expected returns that remains positive but which is not contemporaneous. In other words, the uncertainty effect associated with illiquidity is a dynamic effect that may hide or delay, in the short run, the theoretically positive relationship between expected stock returns and illiquidity, which may be viewed as a long-term or equilibrium relationship. Second, we rely on managed portfolios in our study and not on passive ones—that is, portfolios built using individual stocks that are usually used to analyze the impact of market illiquidity on expected returns. In this special case of *managed* portfolios, like those of hedge funds, the manager can try to capture the illiquidity premium, especially during crises, since risk premia are low or nonexistent in normal times when financial uncertainty is low, and the positive sign associated with the liquidity measures does not reflect a passive adjustment of expected returns to illiquidity shocks but an intended move by portfolio managers. Thus, the sign of the illiquidity measure is subject to interpretation in this case.

**Table 4:** Sensitivities of hedge fund strategies’ returns to *IML* using pooled regressions with fixed coefficients and SUR, 1995–2016

	*IML*				
	Crises	Outside crises	Subprime	Outside subprime	Whole sample
General index	**0.11**	0.03	**0.10**	**0.04**	**0.09**
	***3.58***	*1.31*	***2.17***	***1.71***	***4.02***
Convertibles	**0.17**	− 0.62	**0.26**	− 0.03	**0.09**
	***3.37***	− *0.02*	***4.55***	− *0.96*	***2.91***
Distressed securities	**0.14**	− 0.02	**0.17**	− 0.01	**0.07**
	***4.48***	− *0.76*	***3.73***	− *0.54*	***3.33***
Event driven	**0.11**	0.02	**0.12**	0.02	**0.08**
	***3.35***	*0.64*	***2.63***	*0.67*	***3.40***
Equity market neutral	0.01	− 0.03	0.01	− **0.05**	0.01
	*0.02*	− *1.18*	*0.33*	− ***2.24***	*0.07*
Fixed income	0.01	− **0.04**	0.05	− **0.07**	0.01
	*0.49*	− ***1.65***	*0.99*	− ***2.71***	*0.05*
Futures	− **0.15**	− 0.09	− 0.10	− **0.15**	− **0.10**
	− ***2.01***	− *1.21*	− *1.08*	− ***2.30***	− ***2.01***
Growth	**0.19**	**0.13**	**0.23**	**0.11**	**0.17**
	***2.66***	***1.89***	***2.41***	***1.85***	***3.39***
Long-short credit	0.01	− **0.05**	0.03	− **0.06**	− 0.01
	*0.65*	− ***1.91***	*0.62*	− ***2.59***	− *0.01*
Macro	0.05	0.06	0.03	0.04	**0.07**
	*1.12*	*1.35*	*0.54*	*1.15*	***2.23***
Mergers	**0.04**	0.01	**0.06**	0.01	**0.03**
	***2.53***	*0.48*	***5.36***	*0.79*	***2.45***
Multistrategy	**0.12**	0.01	**0.11**	0.03	**0.08**
	***3.81***	*0.14*	***2.19***	*0.96*	***3.43***
Opportunity index	**0.13**	**0.09**	**0.16**	**0.06**	0.12
	***2.83***	***2.11***	***2.61***	***1.72***	*0.79*
Short-sellers	− **0.27**	− **0.25**	− 0.18	− **0.32**	− **0.24**
	− ***1.88***	− ***1.78***	− *1.01*	− ***2.61***	− ***2.49***
Value index	**0.15**	0.08	0.08	**0.11**	**0.13**
	***2.52***	*1.28*	*1.02*	***2.13***	***2.99***
All strategies	**0.06**	− 0.75	**0.08**	− 0.01	**0.04**
	***3.13***	− *0.39*	***2.30***	− *0.92*	***4.43***

This table provides the estimation of Eq. (18) using pooled regressions accounting for the interaction between strategies’ innovations (SUR, i.e., seemingly unrelated regressions, Zellner 1962). Eq. (18) is estimated over the whole sample period and over our two scenarios described in "Robustness check: response of hedge fund strategies to illiquidity shocks" section. In order to avoid overloading this table, we only report the estimated coefficients associated with *IML* (Pástor and Stambaugh 2003, 2019) for each strategy. t-statistics are in italics. Shaded coefficients are significant at the 10% level or less

The positive exposure to the Pástor-Stambaugh illiquidity measure (*IML*) of the convertibles and distressed securities strategies’ returns during crises—equal to 0.17 and 0.14, respectively, significant at the 1% level, compared to the average market beta of 0.25 and 0.38 (Table 2)—can be linked to the illiquid portfolios they hold (see Table 4). This interpretation is consistent with the high serial correlation of these strategies’ returns—a proxy for illiquidity—that signals illiquid portfolios (see Table 3). Other strategies that have a relatively low serial correlation (i.e., growth, value index, opportunity index, mergers, multistrategy, and event-driven) seem to capture the illiquidity premium during crises, and this interpretation is consistent with their business lines, such as arbitrage. They earn part of their expected returns from bearing illiquidity risk (Hasanhodzic and Lo 2007). Note that the opportunistic strategy, and especially the growth strategy, display positive and significant *IML* loadings outside crises, suggesting that they are particularly successful in capturing these premia. According to Campbell et al. (2010, 2018), growth stocks are good performers during crises, especially vis-à-vis value stocks. They embed real options related to growth opportunities that appreciate when market volatility, which is directly related to market illiquidity, increases. The fact that growth stocks capture illiquidity premia, particularly during crises, could help explain the relatively good performance of this kind of stock during bad times. Finally, the coefficients of the illiquidity measure are significantly negative for some strategies (i.e., futures and especially short sellers). Hence, the short sales in which both strategies are particularly involved seem to be hampered by the illiquidity of financial markets. Interestingly, the exposure of short sellers to *IML*, at − 0.25 outside crises, remains close to this value during crises (− 0.27), which strengthens our conjecture that illiquidity is especially harmful for short sales. Indeed, according to Criton and Scaillet (2011), the liquidity factor is one driver of this strategy.^33^ Surprisingly, the coefficient of the fixed income strategy is also negative at the 10% level outside crises, but it is not significant during crises. As argued previously, for this strategy, uncertainty related to an increase in illiquidity seems to absorb the illiquidity premium (Acharya and Pedersen 2005; Konstantopoulos 2016).

Overall, when no cross-section-specific coefficients are used for the pooled regression with crises,^34^ the common coefficient of *IML* is positive at 0.06 and significant at the 1% level during crises. Thus, the coefficient of *the IML* has the expected positive sign during crises. It is also quite high given the low average level of hedge fund strategies’ betas. However, this loading is not significant outside the crisis. According to Pástor and Stambaugh (2019), it is difficult to estimate liquidity premia outside crises, when the level of uncertainty is much lower.

As expected, illiquidity loadings are usually more important during the subprime crisis for many strategies than during the crises that occurred during our sample period (see Table 4). For instance, while the sensitivity of the growth strategy to *IML* is equal to 0.19 over all crises, it increases to 0.23 during the subprime crisis. The corresponding values for the convertibles strategy are 0.17 and 0.26. Indeed, this strategy earns a non-negligible portion of its expected returns from the illiquidity premium because the firms issuing convertible bonds, which aim to lower the coupon rate, usually have a low credit rating. However, short sellers and futures do not display a significant response to *IML* during the subprime crisis, while this response is significant and substantial over all crises. These two strategies seem to have been immunized against illiquidity shocks during the subprime crisis, which may be associated with the learning process. Interestingly, the fixed income strategy returns do not respond significantly to *IML* during the subprime crisis, even if a large portion of its portfolio was composed of mortgage backed securities (MBS), a major driver of the subprime crisis, and thus a very illiquid security during this crisis. In addition to the overpricing of the MBS during the subprime crisis, another explanation for this lack of response may be the evolution of the fixed income strategy’s beta during the crisis. Its beta registered a substantial increase, further exposing it to negative market returns. Finally, note that, again, when no cross-section-specific coefficients for *IML* is used, the coefficient (0.08) of this variable is positive and significant at the 5% level for all strategies but is insignificant when estimated outside the subprime crisis. Thus, this crisis seems to weigh heavily in the estimation of the illiquidity premium over our sample period.

#### Impact of IML on strategies’ conditional market betas

Table 5 reproduces the experiment presented in Table 4 for the strategies’ conditional market betas. Betas are less responsive to *IML* than are returns. Some strategies, especially those whose portfolios are quite illiquid according to the high serial correlation of their returns, are induced to moderately decrease their beta following a rise in *IML* during crises. These strategies include convertibles, distressed, fixed income, long-short credit, event-driven, and equity market neutral. They engage in market-timing activities during crises aimed at mitigating the impact of rising illiquidity on systematic risk. These strategies, which earn part of their expected returns from bearing illiquidity risk, may seek to protect their returns from market risk, as measured by beta. Given the nature of their operations, they may also be quite sensitive to the uncertainty effect associated with an increase in illiquidity (Acharya and Pedersen 2005; Konstantopoulos 2016). Outside crises, since illiquidity shocks are much lower, fewer strategies decrease their beta after such shocks. Only distressed securities, event-driven, and equity market neutrals reduce their beta following a rise in *IML*. The fact that the equity market neutral strategy is involved in market timing both during crises and outside them suggests that illiquidity is a non-negligible source of risk for this strategy. Consistent with the behavior of its beta after a VIX shock,^35^ such a strategy has difficulty remaining neutral when market illiquidity and volatility trend upward (Criton and Scaillet 2011). The same strategies whose betas display a negative response to *the IML* during all crises also show negative beta responses during the subprime crisis. This response is usually greater, especially for the fixed income strategy, whose sensitivity to *IML* is twice as high during the subprime crisis as is that of all others. This may explain why the return of the fixed-income strategy showed no significant response to *IML* during the subprime crisis.

**Table 5:** Sensitivities of hedge fund strategies’ betas to *IML* using pooled regressions with fixed coefficients and SUR, 1995–2016

	*IML*				
	Crises	Outside crises	Subprime	Outside subprime	Whole sample
General index	− 0.09	− 0.10	− **0.03**	− 0.01	− **0.02**
	− *0.74*	− *0.85*	− ***1.86***	− *1.13*	***2.00***
Convertibles	− **0.07**	− 0.33	− **0.10**	− **0.04**	− **0.06**
	− ***2.53***	− *1.21*	− ***2.95***	− ***1.85***	− ***2.95***
Distressed securities	− **0.04**	− **0.28**	− **0.05**	− **0.04**	− **0.04**
	− ***2.68***	− ***1.88***	− ***2.08***	− ***3.04***	− ***3.50***
Event driven	− **0.04**	− **0.27**	− 0.02	− **0.05**	− **0.04**
	− ***2.40***	− ***1.77***	− *0.76*	− ***3.75***	− ***3.11***
Equity market neutral	− **0.03**	− **0.26**	− 0.01	− **0.05**	− **0.04**
	− ***1.71***	− ***1.67***	− *0.47*	− ***3.26***	− ***2.68***
Fixed income	− **0.03**	− 0.16	− **0.06**	− **0.02**	− **0.03**
	− ***2.78***	− *1.49*	− ***4.09***	− ***2.20***	− ***4.11***
Futures	− 0.03	− 0.32	− 0.02	− **0.04**	− **0.04**
	− *0.88*	− *1.09*	− *0.59*	− ***1.74***	− ***1.68***
Growth	− 0.04	0.02	0.05	− 0.01	− 0.02
	− *0.87*	*0.41*	*0.77*	− *0.14*	− *0.52*
Long-short credit	− **0.02**	− 0.01	− **0.03**	− **0.02**	− **0.02**
	− ***1.70***	− *1.10*	− ***1.64***	− ***2.22***	− ***2.47***
Macro	− 0.01	− 0.01	− 0.04	− 0.01	− 0.01
	− *0.10*	− *0.19*	− *0.78*	− *0.07*	− *0.46*
Mergers	0.00	0.00	**0.02**	0.00	− 0.01
	*0.91*	*0.04*	***1.86***	*0.65*	− *0.51*
Multistrategy	0.02	0.02	− 0.03	0.03	0.01
	*0.61*	*0.63*	− *0.63*	*1.08*	*0.59*
Opportunity index	− 0.05	0.01	− 0.05	− 0.02	− 0.03
	− *1.43*	*0.23*	− *1.04*	− *0.67*	− *1.09*
Short-sellers	− 0.03	− 0.1	0.07	− 0.11	− 0.03
	− *0.35*	− *1.13*	*0.06*	− *1.47*	− *0.81*
Value index	− 0.02	− 0.04	− 0.09	− 0.01	− 0.07
	− *0.28*	− *0.62*	− *1.30*	− *1.13*	− *1.15*
All strategies	0.02	− **0.05**	− **0.04**	− **0.02**	− **0.02**
	*0.75*	− ***2.41***	− ***3.79***	− ***5.85***	− ***3.93***

This table provides the estimation of Eq. (18) using pooled regressions accounting for the interaction between strategies’ innovations (SUR, i.e., seemingly unrelated regressions, Zellner 1962). Eq. (18) is estimated over the whole sample period and over our two scenarios described in "Robustness check: response of hedge fund strategies to illiquidity shocks" section. In order to avoid overloading this table, we only report the estimated coefficients associated with *IML* (Pástor and Stambaugh 2003, 2019) for each strategy. t-statistics are in italics. Shaded coefficients are significant at the 10% level or less

Overall, when using no cross-section-specific coefficients for *IML* in our pooled regressions, we obtain a coefficient equal to − 0.04 for the subprime crisis and to − 0.02 outside this crisis, both coefficients being significant at the 1% level. Hence, hedge fund strategies tend to reduce their beta when confronted with a liquidity shock, but this reaction is more pronounced during the subprime crisis. Our LP analysis will provide more information on the response of strategies’ betas to illiquidity shocks.

### IRFs of illiquidity shocks computed with LP

In this section, we compute LPs during the subprime crisis for the general index and the growth and fixed income strategies’ conditional betas using illiquidity shocks.^36^ We select a strategy that captures the illiquidity premium (i.e., the growth strategy) and a strategy that is representative of strategies holding illiquid securities (i.e., the fixed income strategy). Indeed, we found in the previous section that strategies that reduce their beta following illiquidity shocks are concentrated in the bond category. Incidentally, the orthonormal loadings computed with a principal components analysis run over strategies’ conditional betas indicate that the fixed income, convertibles, distressed securities, and long-short credit strategies are clustered in the same category.^37^ However, even if the beta of the growth strategy shows no sensitivity to *IML* in our pooled regression, which is associated with a static approach, it can display a significant dynamic response to illiquidity when LPs are used.^38^ Thus, it is important to rely on VAR approaches to capture the dynamics of hedge fund behavior.

To implement our experiments, we rely on the innovation in the aggregate illiquidity measure (labeled *i_gamma*) developed by Pástor and Stambaugh (2003). Note that these authors propose three illiquidity measures: the aggregate liquidity measure, the innovation in aggregate liquidity, and *IML*, which we used in the previous section. The first two measures are non-tradable, while the third is tradable. According to Pástor and Stambaugh (2003, 2019), the tradable measure should be used when estimating an asset pricing model; otherwise, the alpha will be biased. Indeed, they contend that all explanatory variables must be tradable in an asset-pricing model. In all other cases, they recommend relying on *the i_gamma* measure. This measure is free from serial correlation, while the aggregate liquidity measure is not. Indeed, as argued by Franses (2002), neglecting serial correlation in the residuals leads to suboptimal statistical inference and inaccurate out-of-sample forecasts. More precisely, the *i_gamma* measure is obtained by removing the serial correlation from the aggregate liquidity measure. The latter measure, called *gamma*, is computed by running the following regression: $r_{i,d + 1,t}^{e} = \theta_{i,t} + \phi_{i,t} r_{i,d,t} + \left( {gamma} \right)_{i,t} sign\left( {r_{i,d,t}^{e} } \right)\left( {Volume_{i,d,t} } \right) + \varepsilon_{i,d + 1,t}$, where $r_{i,d,t}^{e}$ is the stock excess return ($r_{i,d,t}$) of firm *i* above the CRSP value-weighted market return on day *d* in month *t*, and $Volume_{i,d,t}$ is the dollar volume on this stock on day *d* in month *t*. The marketwide measure of liquidity for month *t* is the average of the gammas computed for this month for all individual stocks included in the CRSP index. Intuitively, *gamma* measures the reverse of the previous day’s order flow shock. *Gamma* (aggregate liquidity) usually has a negative sign. The larger the absolute value of *gamma*, the larger the implied price impact (illiquidity). In this section, we rely on the *i_gamma* measure—that is, the gamma measure corrected for serial correlation and the key series of Pástor and Stambaugh (2003)—since we are not interested in asset pricing models per se but in the response of hedge fund-conditional beta to illiquidity shocks.

In line with Beaudry et al. (2001), we distinguish between risk and uncertainty to determine the impact of illiquidity on the conditional beta. Risk is associated with the *first moment* of a macroeconomic or financial variable proxying for risk (i.e., *i_gamma* in this section). The *second moment* of a macroeconomic or financial variable— that is, its conditional variance—gauges uncertainty. We compute the conditional variance of *i_gamma*, labeled *cv_igamma*, by estimating the variance of the innovation associated with an Autoregressive Moving Average (ARMA) process run over *i_gamma* and estimated using an Exponential GARCH procedure (EGARCH, Nelson 1991). We select this procedure because the behavior of *i_gamma* is asymmetrically dependent on the phase of the business cycle.

**Fig. 4:**
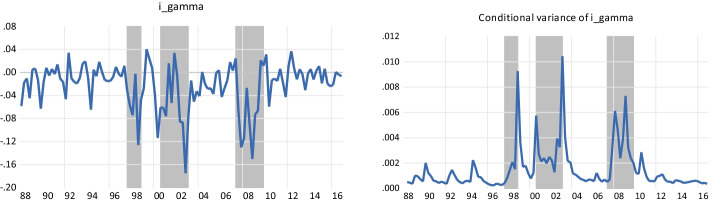
Innovation in aggregate liquidity (*i_gamma*) and conditional variance of i_gamma, 1988Q1-2016Q2. *Notes:* These plots reproduce the innovation in aggregate illiquidity (*i_gamma*), as published by Pástor and Stambaugh (2003), and its conditional variance. The conditional variance is computed using the EGARCH method in order to account for the asymmetries in the behavior of *i_gamma* dependent of the phase of the business cycle. Shaded areas correspond to periods of crises

Figure 4 shows the plots of *i_gamma* and *cv_igamma*. The shaded areas correspond to the crises that occurred during our sample period. We observe that the *i_gamma* measure becomes more volatile during crises. The troughs of this indicator also take place during crises, since it is a liquidity measure.^39^ Peaks in this series may also be observed during crises, such as the tech-bubble one, and they may be associated with liquidity injections by the U.S. Federal Reserve Bank. Hence, *i_gamma* is characterized by its higher volatility more than its lower level during crises. The *cv_igamma* measure—our gauge of liquidity uncertainty—displays behavior over the business cycle that is more asymmetric than that of *i_gamma*: It is relatively low during normal times but tends to jump during crises. However, these jumps are not persistent, since they reverse quickly after one or two quarters. Note that *cv_igamma* may also peak outside major US crises, as is observed in 2010, associated with the European sovereign debt crisis.

Our measure of liquidity uncertainty (*cv_igamma*) is an econometric-generated variable and a potentially noisy proxy for its associated unobservable measure.^40^ Pagan (1984) defines a generated variable as a regressor of a model that is constructed as a prediction of another regression.^41^*cv_igamma* is a generated variable because it is a regressor in our VAR models and is devised using an EGARCH model (i.e., another regression). According to Pagan (1984, 1986), failing to instrument a generated variable to tackle the measurement error embedded in this kind of variable may invalidate the *t-*tests. Pagan and Ullah (1998) even go so far as to say that failing to correct this measurement error may lead to inconsistent estimators. To address this bias, in addition to the classical predetermined explanatory variables of our model (Eq. (15)), we first regress *cv_igamma* on *robust* instruments, which are the higher moments (cumulants) of these explanatory variables—*dlnGDP*, *term_spread*, *credit spread* and *i_gamma*—expressed in deviation from their means (Fuller 1987; Lewbel 1997; Racicot 2015; Racicot and Rentz 2015; Racicot et al. 2018; Racicot et al. 2019). To simplify, we introduce only the square of our explanatory variables in deviation from their means as higher moment instruments in the following equation in order to “instrumentate” *cv_igamma*:

19 $$\begin{gathered} cv\_igamma_{t} = \gamma_{0} + \gamma_{1} d\ln GDP_{t - 1} + \gamma_{2} credit\_spread_{t - 1} + \gamma_{3} term\_spread_{t - 1} + \gamma_{4} i\_gamma_{t - 1} + ... \hfill \\ ... + \gamma_{5} \left( {d\ln GDP_{t} - \overline{d\ln GDP} } \right)^{2} + \gamma_{6} \left( {credit\_spread_{t} - \overline{credit\_spread} } \right)^{2} + \gamma_{7} \left( {term\_spread_{t} - \overline{term\_spread} } \right)^{2} + ... \hfill \\ ... + \gamma_{8} \left( {i\_gamma_{t} - \overline{i\_gamma} } \right)^{2} + \xi_{t} \hfill \\ \end{gathered}$$

where $\overline{x}$ is the average of *x*.

Our gauge of liquidity uncertainty is $\widehat{cv\_igamma}$—that is, the value of *cv_igamma* predicted by Eq. (19).

Before examining the results, it is important to examine the link between market volatility, as measured by the VIX, and market illiquidity. We expect a positive correlation between these two variables. When financial markets become more volatile, they should also become more illiquid. However, this conjecture needs to be qualified. Indeed, Adrian et al. (2017) argue that moderate increases in market liquidity may result in higher volatility, while large increases in market volatility may entail a substantial drop in the liquidity of financial markets. This argument is in line with that of Campbell et al. (2018), who assert that there is no reason to assume that higher volatility always reduces aggregate stock prices. They refer to the stock boom of the 1990s, when stock prices were both high and volatile. Hence, the relationship between market volatility and market liquidity may depend on the phase of the business cycle.

We use five indicators to gauge market liquidity (illiquidity): (i) the Pástor and Stambaugh (PS) (2003, 2019) non-tradable measure of liquidity corrected for autocorrelation, (ii) an uncertainty measure of illiquidity built using the first measure, (iii) the Pástor and Stambaugh (PS) (2003, 2019) tradable illiquidity measure (*IML*), (iv) the Amihud (2002, 2019) ratio,^42^ and (v) the term spread. The first indicator is a measure of liquidity, in the sense that an increase in this indicator signals a rise in market liquidity. Other indicators are barometers of the degree of financial market illiquidity. Over our whole sample, with a coefficient of 0.58, our uncertainty measure is the most correlated with the VIX, and this correlation (0.74) is even higher during the subprime crisis. These correlations suggest that the co-movements between the VIX and market illiquidity are asymmetric. The Amihud ratio illiquidity measure (0.45) and the PS non-tradable liquidity measure (− 0.34) are also significantly correlated with the VIX over our entire sample period with the expected signs. However, the *IML* illiquidity measure has an (unexpected) negative correlation (− 0.11) with the VIX, while there is no obvious link between the term spread and the VIX over the whole sample. However, these correlations depend on the stance of the business cycle. For instance, during the subprime crisis, the coefficients of correlation of *IML* and the term spread with the VIX were − 0.50 and 0.48, respectively.^43^ The term spread may thus be closely correlated with the VIX during a crisis, while the negative correlation between *IML* and VIX may even increase. Overall, these results suggest that market illiquidity has many dimensions that are not fully captured by the VIX. They may also suggest that an increase in market volatility is not necessarily an impediment to the functioning of financial markets.

**Fig. 5:**
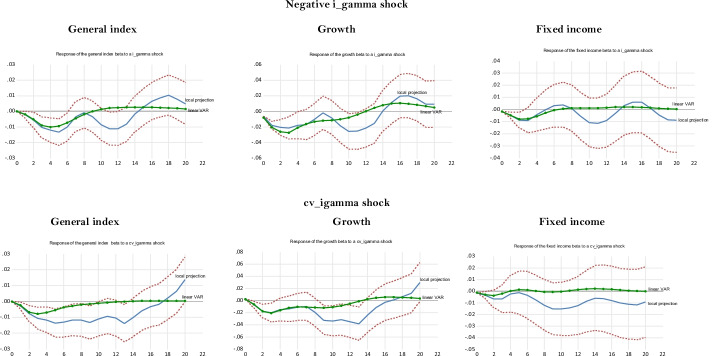
Local projection of illiquidity shocks on the beta of the general index, growth and fixed income strategies over the subprime crisis with *cv_igamma* instrumented. *Notes:* Liquidity shocks are measured by the main series of Pástor and Stambaugh (2003)—i.e., the innovation in aggregate liquidity (*i_gamma*)—and its conditional variance as computed by the EGARCH (Nelson 1991) method (*cv_igamma*). The impulse response functions corresponding to the linear LP are built using the Jordà’s (2004, 2005, 2009) algorithm provided by Eren Ocakverdi, consultant at Yapy Kredi. The LP of a beta strategy is performed over the subprime crisis (2007Q3–2009Q4). We also provide the linear VAR estimated over the whole sample period selected as benchmark. The confidence interval of the LP impulses, computed at the threshold of 95%, is in dotted lines. As explained in the paper, our model comprises six endogenous shocks, placed in the following order to perform the Cholesky factorization (Sims 1980) required to build structural shocks: GDP growth, term spread, credit spread, beta (own shock), *i_gamma*, and *cv_igamma*. As usual, these variables are ordered by increasing level of endogeneity. This order relies on the conventional information criteria: Akaike and Schwartz criteria

We now turn to the LPs of the conditional betas for the general index and for the growth and fixed income strategies over the course of the subprime crisis using our illiquidity measures (see Fig. 5). To perform our experiments, we substitute *i_gamma* and our instrumented measure of *cv_igamma* with VIX in Eq. (15); VIX is significantly correlated with these two variables, as shown earlier. *Negative i_gamma* shocks (i.e., illiquidity shocks) are used to conduct the experiments.

The linear VAR indicates that the general index beta declines on average after a negative *i_gamma* shock (i.e., a shock associated with liquidity risk). This is the expected move if hedge funds monitor liquidity risk. However, this decrease is short-lived, as the beta progressively returns to its initial value after several quarters. Using LP over the subprime crisis, we observe a decrease in beta, which is much more persistent than that observed with the linear VAR. Moreover, the fluctuations in the beta suggest that, when the general index beta tends to rise, hedge funds react by monitoring it downward. The beta of the growth strategy follows the same profile as that of the general index after a negative *i_gamma* shock, although the decline computed with LP is more important. This is normal because the growth strategy is the most procyclical in our sample. For its part, the beta of the fixed income strategy also decreases following an *i_gamma* shock, and this drop becomes more persistent with the LP method. The fluctuations of its beta during the subprime crisis suggest that it was difficult to control its systematic risk. Indeed, it suffered greatly from the collapse of the mortgage-backed securities market.

Turning to a *cv_igamma* shock (i.e., a shock associated with liquidity uncertainty) the general index beta decreases weakly after this shock when the linear VAR is used. This shows a slow recovery after the shock. By contrast, when using LP, we observe a significant decrease in beta, which is much more important than that associated with the *i_gamma* shock. Moreover, this drop was persistent. Once more, the beta profile of the growth strategy is similar to that of the general index, but the decline is more pronounced, as expected.^44^ In line with the general index, the beta of the growth strategy responds more to a *cv_igamma* shock than to an *i_gamma* shock. Finally, the beta of the fixed income strategy also responds negatively to a *cv_igamma* shock, but this move is not significant. Therefore, this strategy seems to have experienced great difficulty in coping with the uncertainty generated by the illiquidity of financial markets during the subprime crisis.

Overall, the second moment of liquidity, a neglected variable in the financial literature, explains the response of hedge funds to liquidity conditions prevailing in financial markets. In our study, the impact of *cv_igamma* is more important for the behavior of hedge funds than is that of the first moment of liquidity (i.e., *i_gamma*). Our findings suggest that hedge funds tended to reduce their beta more when liquidity uncertainty increased than when illiquidity risk tended to increase during the subprime crisis.

**Fig. 6:**
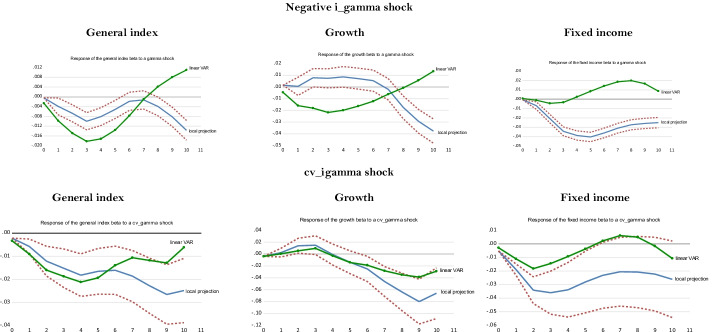
Local projection of illiquidity shocks on the beta of the general index, growth and fixed income strategies over the subprime crisis with *cv_igamma* not instrumented. *Notes:* Liquidity shocks are measured by the main series of Pástor and Stambaugh (2003)—i.e., the innovation in aggregate liquidity (*i_gamma*)—and its conditional variance as computed by the EGARCH (Nelson 1991) method (*cv_igamma*). The impulse response functions corresponding to the linear LP are built using the Jordà’s (2004, 2005, 2009) algorithm provided by Eren Ocakverdi, consultant at Yapy Kredi. The local projection of a beta strategy is performed over the subprime crisis (2007Q3–2009Q4). We also provide the linear VAR estimated over the whole sample period selected as benchmark. The confidence interval of the LP impulses, computed at the threshold of 95%, is in dotted lines. As explained in the paper, our model comprises six endogenous shocks, placed in the following order to perform the Cholesky factorization (Sims 1980) required to build structural shocks: GDP growth, term spread, credit spread, beta (own shock), *i_gamma*, and *cv_igamma*. As usual, these variables are ordered by increasing level of endogeneity. This order relies on the conventional information criteria: Akaike and Schwartz criteri

Because we have corrected *cv_igamma*—a generated variable—for its error-of-measurement bias, it is worthwhile investigating whether our IRFs change when we rely on the uncorrected *cv_igamma* variable to run our experiments. The corresponding IRFs are shown in Fig. 6. The results are much less coherent than those obtained using $\widehat{cv\_igamma}$. Regarding the negative *i_gamma* shock, we first note in Fig. 6 that the decrease in the general index beta is more important with the linear VAR, which suggests that hedge funds responded more, on average, to an *i_gamma* shock than they did during the subprime crisis, which is not very convincing. Second, for the growth strategy, the IRFs computed with the two methods go in the opposite way: a substantial decrease in the beta with the linear VAR and an increase during the subprime crisis. These opposing moves are not intuitive. Why do hedge funds reduce their beta on average when illiquidity risk is quite normal and act in the opposite way during the subprime crisis when liquidity risk peaks? This behavior is puzzling. Third, the response of the fixed income strategy’s beta to an *i_gamma* shock is particularly bizarre. Indeed, in Fig. 6, we observe a large drop in its beta with a very tight confidence interval when LP is used, whereas the opposite trend is seen when the linear VAR is used.

Turning to the responses of strategies’ betas to the uncorrected *cv_igamma*, we note an increase in the growth strategy’s beta after such a shock with the two econometric methods used. This behavior is difficult to explain because this strategy is the most procyclical in our sample, especially because it weighs heavily in the general index whose beta responds in the opposite way to this kind of shock. Moreover, the good monitoring of illiquidity uncertainty performed by the fixed income strategy when the rough measure *cv_igamma* is used, especially during the subprime crisis, is questionable because of the great difficulties this strategy encountered during this crisis.

Failing to correct *cv_igamma* for its error-of-measurement bias may thus lead to spurious IRFs in a VAR analysis. This can contaminate not only the IRFs of the generated variable, *cv_igamma*, but also the IRFs of the other variables of the model (here *i_gamma*). Thus, it appears important to “instrumentate” generated variables before introducing them into a VAR analysis.

## Policy implications

In this section, we discuss the policy implications of our findings, focusing on monetary policy and financial stability.

Aside from being an indicator of market liquidity, the term spread is also an indicator of monetary policy, which has had a growing influence during our period of analysis given the downward trend in interest rates. Indeed, in an environment characterized by zero short-term rates like the current one, the Fed uses the term spread, among other tools, to ease credit conditions.^45^ To see how it operates, let us rewrite the equation of the term spread:

20 $${\text{Term spread }} = r_{lt} - r_{st} = E_{t} \left[ {\frac{1}{m}\sum\limits_{j = 0}^{m - 1} {r_{s,t + j} } } \right] + \phi_{tm} - r_{st}$$

According to Eq. (20), the central bank can influence the term spread inasmuch as it can impact expectations related to short-term rates. Central banks communicate their intentions about the path of future short rates (Gertler and Karadi 2015). However, our findings show that a term spread shock has less impact on the risk-taking of hedge fund strategies than other kinds of shocks, such as VIX shocks and GDP shocks. According to Nelson et al. (2015), Buchak et al. (2017), and Farhi and Tirole (2018), shadow banks, which include hedge funds, seem to constitute a loophole in the transmission channel of monetary policy.

In "Robustness check: response of hedge fund strategies to illiquidity shocks" section, we found that hedge fund strategies respond more to liquidity uncertainty than to liquidity risk. This reaction is very nonlinear because it is much more important during crises than during normal times. According to Eq. (5), the substantial decrease in the beta of hedge fund strategies after a shock related to liquidity uncertainty corresponds to an important deleveraging by hedge funds. This deleveraging process may be accompanied by massive “fire sales” of financial assets, which may destabilize financial markets (Brunnermeier and Pedersen 2009; Shleifer and Vishny 2010; Brunnermeier and Sannikov 2014). Monetary policy is obviously concerned with liquidity uncertainty. When this kind of uncertainty arises, central banks must demonstrate a strong will to restore normal levels of liquidity in financial markets. To achieve this, they must develop liquidity uncertainty measurement tools similar to the one we used to implement our local projections. Central banks should apply these tools to other financial institutions, especially commercial and investment banks.

**Fig. 7:**
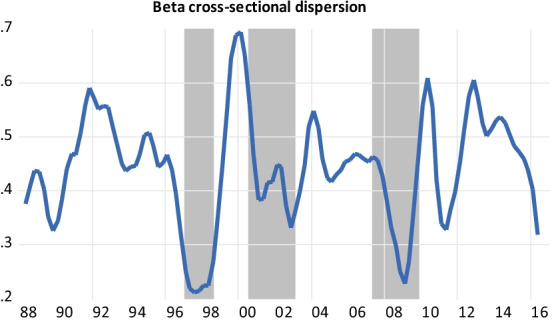
Beta cross-sectional dispersion of hedge fund strategies. *Notes:* For every month, we compute the standard deviation of the conditional strategies’ betas built with the MGARCH procedure. Crisis episodes are shaded

Our findings suggest that the response of hedge fund strategies to macroeconomic and financial shocks is quite homogeneous during crises, especially for shocks associated with market liquidity and market volatility. Indeed, as argued in "Stylized facts"  section, most hedge funds are in the short volatility category. Since our findings suggest that hedge fund strategies respond more to VIX shocks than to GDP growth shocks, this is a source of homogeneity in the behavior of hedge fund strategies, especially during crises when VIX shocks dominate. However, this homogeneity is a source of systemic risk, particularly during turmoil (Beaudry et al. 2001; Baum et al. 2009; Wagner 2010). A plot of the beta cross-sectional dispersion over time allows us to track the evolution of homogeneity (heterogeneity) in hedge funds’ risk behavior in order to capture periods of greater systemic risk (Beaudry et al. 2001; Baum et al.; Caglayan and Xu 2016a, b). Therefore, for every month in our sample, we compute the standard deviation of the strategies’ time-varying (conditional) beta (see Fig. 7):

$${\text{cross - sectional dispersion = }}\frac{1}{n - 1}\sum\limits_{i = 1}^{n} {\left( {\beta_{it} - \overline{\beta }_{it} } \right)}^{2} \quad \forall t$$

where *n* is the number of strategies (i.e., 14 in this study; see Table 1), and $\overline{\beta }_{it}$ is the average of the strategies’ betas for month *t*.

We note that the risk behavior of hedge funds in terms of beta becomes more homogeneous during crises, as the beta cross-sectional dispersion drops and hits its lows during the three crises identified in Fig. 7 (i.e., Asian-Russian-LTCM, tech-bubble, and subprime crises). The decrease in the cross-sectional dispersion was particularly important during the 1997–1998 crisis, when a major hedge fund (i.e., LTCM) collapsed. Thus, the hedge fund industry may be a source of substantial systemic risk that can threaten financial stability. Our findings are similar to those of Ang et al. (2011), who use leverage rather than beta to monitor risk in the hedge fund industry. They find that VIX is a major driver of leverage and that hedge fund leverage decreases when VIX increases. As in our analysis, they find that leverage decreased markedly during the subprime crisis, while that of most financial intermediaries, especially investment banks, soared. As argued by Ang et al. (2011), this commonality in the behavior of hedge funds may create a fire-sale externality that causes systemic risk.^46^ In this context of rising systemic risk in the hedge fund industry during crises, short sales performed by short sellers and futures strategies for delivering positive payoffs appear detrimental to financial stability. Indeed, short positions are a form of leverage, since downside risk is theoretically unlimited (McGuire et al. 2005).

To better grasp the drivers of the beta cross-sectional dispersion, we transpose our benchmark beta model given by Eq. (15) for this variable. We rely on two versions of this model. Model 1 includes the VIX but excludes our liquidity measures (i.e., *i_gamma* and *cv_igamma*) since, as shown above, these variables are closely correlated:

21 $$cs\_disp\_beta_{t} = \gamma_{0} + \gamma_{1} d\ln GDP_{t} + \gamma_{2} credit\_spread_{t} + \gamma_{3} term\_spread_{t} + \gamma_{4} VIX_{t} + \varepsilon_{it}$$

where *cs_disp_beta*_*t*_ is the beta cross-sectional dispersion observed at time *t*.

In model 2, we substitute our liquidity measures with VIX in Eq. (21):

22 $$cs\_disp\_beta = \varphi_{0} + \varphi_{1} d\ln GDP_{t} + \varphi_{2} credit\_spread_{t} + \varphi_{3} term\_spread_{t} + \varphi_{4} i\_gamma + \varphi_{5} \widehat{cv\_igamma} + \varepsilon_{it}$$

where $\widehat{cv\_igamma}$ is the instrumented value of *cv_igamma*.

**Fig. 8:**
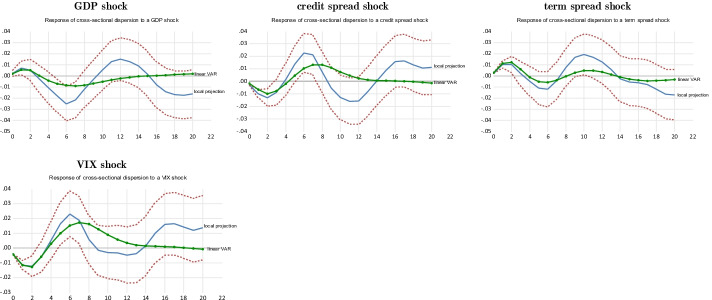
Response of the beta cross-sectional dispersion to shocks, model 1. *Notes:* Model 1 is given by equation (21). The impulse response functions corresponding to the linear LP—i.e., the beta cross-sectional dispersion forecast following external shocks—are built using the Jordà’s (2004, 2005, 2009) algorithm provided by Eren Ocakverdi, consultant at Yapy Kredi. The local projection of the beta cross-sectional dispersion is performed over the subprime crisis (2007Q3–2009Q4). We also provide the linear VAR estimated over the whole sample period selected as benchmark. The confidence interval of the LP impulses, computed at the threshold of 95%, is in dotted lines

Figure 8 shows the LP of Model 1 (Eq. (21)) during the subprime crisis. Consistent with our previous findings, the VIX shock is the major driver of the homogenous behavior of hedge fund strategies during the subprime crisis. Indeed, the beta cross-sectional dispersion significantly decreased after this shock. Hence, market volatility is a major source of systemic risk in the hedge fund industry. The credit spread is another driver of the homogenous behavior of hedge fund strategies. These results are in line with Billio et al. (2012), who find that credit spread and VIX are common factors in the hedge fund industry, especially during crises.^47^ By contrast, a GDP shock contributes to the heterogeneous behavior of hedge fund strategies. This is consistent with our analysis of procyclical and countercyclical strategies, which are well-balanced in the hedge fund industry. This is also the case for the term spread shock, where the beta cross-sectional dispersion increases at the impact of this shock. As explained above, the term spread is a countercyclical indicator, in addition to being an illiquidity indicator, and these two distinct dimensions may explain the heterogeneous behavior of hedge fund strategies vis-à-vis this shock. Strategies that rely the most on money markets to finance their portfolios suffer the most from an increase in the term spread, while others may benefit as they seek to capture this spread.

**Fig. 9:**
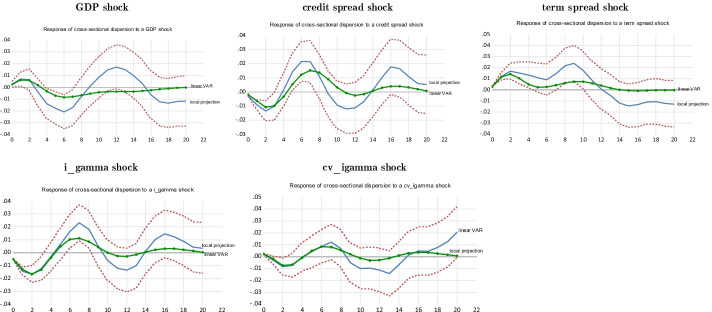
Response of the beta cross-sectional dispersion to shocks, model 2. *Notes:* Model 2 is given by equation (22). The impulse response functions corresponding to the linear LP—i.e., the beta cross-sectional dispersion forecast following external shocks—are built using the Jordà’s (2004, 2005, 2009) algorithm provided by Eren Ocakverdi, consultant at Yapy Kredi. The local projection of the beta cross-sectional dispersion is performed over the subprime crisis (2007Q3–2009Q4). We also provide the linear VAR estimated over the whole sample period selected as benchmark. The confidence interval of the LP impulses, computed at the threshold of 95%, is in dotted lines

Figure 9 shows the LP of Model 2 (Eq. (22)) during subprime crisis. The responses of the beta cross-sectional dispersion to GDP, credit spread, and term spread shocks are similar to those obtained with Model 1. Regarding our two illiquidity measures, we note that both *i_gamma*^48^ and *cv_igamma* contribute to the homogenous behavior of hedge funds during the subprime crisis. This finding is consistent with our previous results, where we observed that hedge funds tend to reduce their beta after a negative *i_gamma* shock but also following a *cv_igamma* shock. Both liquidity risk and liquidity uncertainty are thus sources of systemic risk in the hedge fund industry.

Our study also has implications for analyses of the impact of the COVID-19 pandemic on the performance of hedge funds. Even if this crisis seems to be less stressful for hedge funds than the subprime crisis, it has still caused a substantial decrease in their assets under administration (AUM) and a concomitant massive increase in their redemptions at the start of the crisis.^49^ In September 2020, new rules were introduced for EU-domiciled hedge funds,^50^ according to which they must regularly test their resilience to market and liquidity risks and report their findings to local regulators. The indicators and tools we propose, especially those for analyzing liquidity risk and uncertainty, may be quite useful in developing these resilience tests.

## Conclusion

This study investigates how hedge fund strategies market-time their systematic risk under extreme scenarios, especially during the subprime crisis, which has been the worst since the Great Depression. Using a time-varying measure of beta (i.e., conditional beta computed with the MGARCH process), we first *forecast* the market timing of strategies during the subprime crisis by relying on a LP algorithm using a model designed to predict the responses of hedge fund strategies to macroeconomic and financial shocks, especially VIX shocks and illiquidity shocks. Using TVAR, we study^51^ the response of strategies’ betas to a combination of moderate and strong macroeconomic and financial shocks—positive and negative—defined over two extreme regimes. With the help of LP and TVAR, we present four notable findings.

First, the asymmetries in the responses of hedge fund strategies’ betas are much higher during the subprime crisis than during normal times, as measured by the differences between the amplitude of the IRFs computed with LP over the subprime crisis and those built with a benchmark VAR, which provides an “average” reaction over the whole sample period. These asymmetries are particularly important for procyclical strategies following VIX shocks, the VIX measuring market volatility, and investor fear. This kind of shock induces them to substantially reduce their beta—that is, a deleveraging process.^52^ Thus, VIX shocks should be mainly responsible for the volatility of the beta of procyclical strategies. GDP growth shocks have a smaller impact, perhaps because market volatility as given by the VIX is easier to forecast than GDP growth. Indeed, in contrast to GDP growth, market volatility tends to persist, and it is predictable to some extent (Busse 1999). The beta of two countercyclical strategies—short sellers and futures—increased during the subprime crisis quite asymmetrically relative to normal times. These strategies would appear to have benefited from the subprime crisis owing to their market timing skills.

Second, we examine the impact of illiquidity risk and illiquidity uncertainty on hedge fund strategies’ conditional betas. Since illiquidity premiums are low in normal times, we focus our analysis on crises or recessions. We innovate by introducing a measure of illiquidity uncertainty computed with an EGARCH procedure in the analysis of hedge fund market timing activities. We find that hedge fund managers are involved in illiquidity timing during crises and that their beta responds more to illiquidity uncertainty than to illiquidity risk. We show that it is important when conducting such an analysis to correct the generated variables (i.e., illiquidity uncertainty) for error-of-measurement bias (Pagan 1984, 1986; Pagan and Ullah 1988). To the best of our knowledge, no hedge fund researcher has performed such a correction to a generated illiquidity variable.

Third, by transposing our benchmark model to the analysis of the beta cross-sectional dispersion, we found that the VIX, our two measures of illiquidity, and credit spread are major drivers of systemic risk in the hedge fund industry. Thus, hedge fund strategies tend to adopt homogenous behavior vis-à-vis these risk factors. Adverse shocks associated with these factors tend to deleverage in unison, which may lead to fire sales, a major source of systemic risk (Brunnermeier and Pedersen 2009; Shleifer and Vishny 2010; Brunnermeier and Sannikov 2014).

Finally, the responses of procyclical and countercyclical strategies to shocks may be quite different. An adverse GDP growth shock may be detrimental to procyclical strategies—that is, they may struggle to control a strong shock as opposed to a moderate one—while countercyclical strategies may benefit from such a shock. These latter strategies thus seek to capture the risk premia or spreads between their long and short positions on securities (i.e., volatility, credit, and illiquidity risk premia). These non-traditional sources of risk may be very attractive to investors, especially during turmoil (Hasanhodzic and Lo 2007). Two countercyclical strategies that are particularly involved in short sales—short sellers and futures—benefit from a positive VIX shock, especially in the low regime. They increase their exposure to volatility in this regime by leveraging their beta to take advantage of the falling stock markets.

Overall, our experiments show that it is important to rely on dynamic methods (i.e., LP and TVAR) to track the behavior of managed portfolios such as those held by hedge funds. Static approaches like those we used to examine the impact of illiquidity shocks on the behavior of hedge funds may indicate that strategies are insensitive to illiquidity risk or uncertainty shocks, while our dynamic approaches signal that this behavior may be quite complex and time-varying. We find that the uncertainty effect associated with illiquidity is a dynamic dimension that may mitigate, at least in the short run, the theoretically positive relationship between expected stock returns and illiquidity—that is, a long-term or equilibrium relationship. To the best of our knowledge, we are the first to conduct this kind of dynamic analysis using an indicator of liquidity uncertainty, which suggests that the second moment related to illiquidity really matters. Further research is required on this issue to enhance our grasp of the market timing activities of hedge fund managers, especially during crises, when liquidity risk, liquidity uncertainty, and volatility risk peak.

## Figures and Tables

**Fig. 10:**
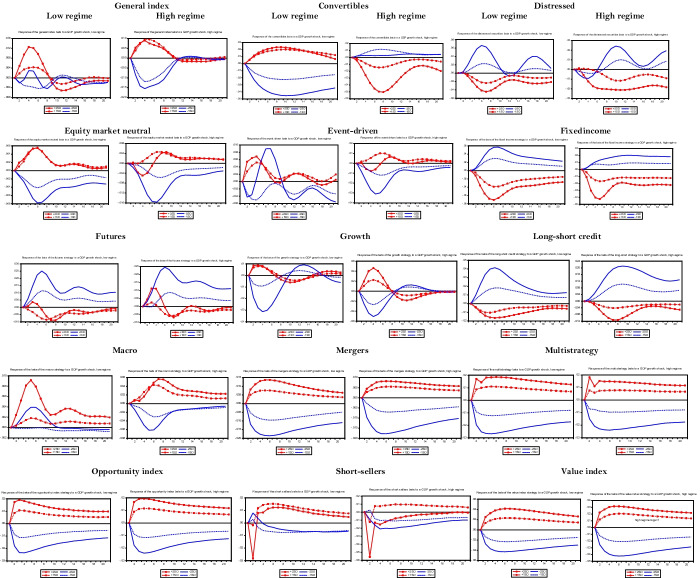
Response of hedge fund beta to a moderate and strong negative and positive GDP growth shocks in low and high regimes, general index and strategies. *Notes:* These plots correspond to the impulse response functions of strategies’ betas to GDP growth shocks. They are built with the Balke’s (2000) nonlinear impulse response functions algorithm using the generalized impulse response function to identify structural shocks (Koop et al. 1996; Pesaran and Shin 1997, 1998). For each strategy, we have two kinds of shocks: positive and negative shocks of GDP growth, and moderate and strong shocks, corresponding to one and two standard deviations of the value of the shocks, respectively, for a total of four impulse response functions by regime for each strategy. Moreover, Balke (2000) considers two categories of regimes separated by an optimal threshold value: a low and a high regime. The significance of the threshold values is evaluated with Wald tests

**Fig. 11:**
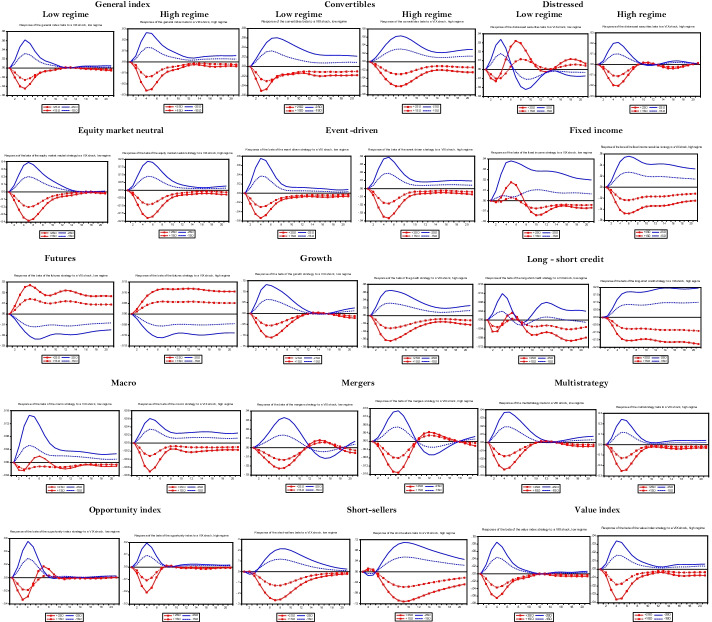
Response of hedge fund beta to moderate and strong negative and positive VIX shocks in low and high regimes, general index and strategies. *Notes:* These plots correspond to the impulse response functions of strategies’ betas to VIX shocks. They are built with the Balke’s (2000) nonlinear impulse response functions algorithm using the generalized impulse response function to identify structural shocks (Koop et al. 1996; Pesaran and Shin 1997, 1998). For each strategy, we have two kinds of shocks: positive and negative VIX shocks, and moderate and strong shocks, corresponding to one and two standard deviations of the value of the shocks, respectively, for a total of four impulse response functions by regime for each strategy. Moreover, Balke (2000) considers two categories of regimes separated by an optimal threshold value: a low and a high regime. The significance of the threshold values is evaluated with Wald tests. We use GDP growth to compute the threshold value separating the regimes

**Fig. 12:**
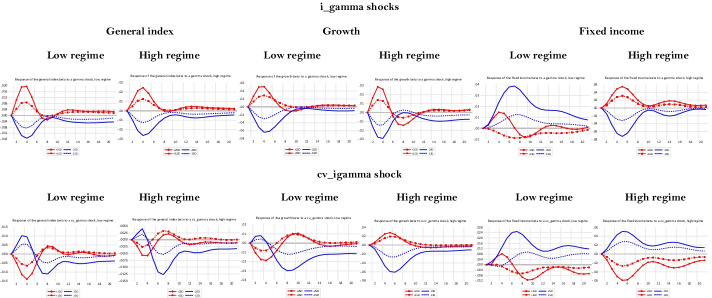
Response of hedge fund beta to a moderate and strong negative and positive illiquidity shocks in low and high regimes for the general index and the fixed income and growth strategies. *Notes:* Liquidity shocks are measured by the main series of Pástor and Stambaugh (2003)—i.e., the innovation in aggregate liquidity (*i_gamma*)—and its conditional variance as computed with the EGARCH (Nelson 1991) method (*cv_igamma*). The plots correspond to the impulse response functions of strategies’ betas to liquidity shocks. They are built with the Balke (2000) nonlinear impulse response function algorithm using the generalized impulse response function to identify the structural shocks (Koop et al. 1996; Pesaran and Shin 1997, 1998). For each strategy, we have two kinds of shocks: positive and negative shocks of liquidity, and moderate and strong shocks of liquidity, corresponding to one and two standard deviations of the value of the shocks, respectively, for a total of four impulse response functions by regime for each strategy. Moreover, Balke (2000) considers two categories of regimes separated by an optimal threshold value: a low and a high regime. The significance of the threshold values is evaluated with Wald tests. We use real GDP growth to compute the threshold value separating the regimes

## Data Availability

Data and materials are available upon reasonable request.

## Appendix 1

### Threshold VAR (TVAR)

#### Method

The structural TVAR model as developed by Balke (2000) reads as follows:

23 $${\mathbf{Y}}_{{\mathbf{t}}} {\mathbf{ = A}}^{1} {\mathbf{Y}}_{{\mathbf{t}}} {\mathbf{ + B}}^{{\mathbf{1}}} {\mathbf{(L)Y}}_{{{\mathbf{t - 1}}}} + \left[ {{\mathbf{A}}^{{\mathbf{2}}} {\mathbf{Y}}_{{\mathbf{t}}} {\mathbf{ + B}}^{{\mathbf{2}}} {\mathbf{(L)Y}}_{{{\mathbf{t - 1}}}} (I(c_{t - d} > \gamma ))} \right] + {\mathbf{U}}_{{\mathbf{t}}}$$

where **Y**_**t**_ is a vector containing the variables which constitute the VAR; **B**^**1**^**(L)** and **B**^**2**^**(L)** are lag polynomial matrices in the two regimes, and **U**_**t**_ are structural innovations. $c_{t - d}$ is the threshold variable that determines in which regime the system is and $I\left( {c_{t - d} } \right) > \gamma$ is an indicator function which takes the value of 1 if $c_{t - d} > \gamma$ and 0 otherwise. **A**^**1**^ and **A**^**2**^ are associated with the structural contemporaneous relationships in regimes 1 and 2.

Since the TVAR given by Eq. (23) is nonlinear, the amplitude of the corresponding IRFs depends on the sign of a shock and varies nonlinearly with the size of this shock—i.e., shocks are non-proportional to size. In this respect, Balke (2000) computes the IRFs corresponding to positive and negative shocks for the two regimes which he *optimally* defines—i.e., a low and a high regime. For each regime, he also defines IRFs associated with two shocks of different size: (i) a moderate (standard shock) equal to one standard deviation of the corresponding innovation (+ 1SD); (ii) a big shock, defined as two standard deviations of the innovation (+ 2SD). As such, the TVAR is particularly well-suited to study the various dimensions of nonlinearities in the behavior of hedge fund strategies’ betas over the business cycle.

As argued by Balke (2000), if the threshold value were known, the significance test of the threshold would be **A**^**2**^ = **B**^**2**^**(L)** = 0 under the null hypothesis. Under H0, Eq. (23) then becomes:

24 $${\mathbf{Y}}_{{\mathbf{t}}} {\mathbf{ = A}}^{1} {\mathbf{Y}}_{{\mathbf{t}}} {\mathbf{ + B}}^{{\mathbf{1}}} {\mathbf{(L)Y}}_{{{\mathbf{t - 1}}}} + {\mathbf{U}}_{{\mathbf{t}}}$$

i.e., a standard linear VAR.

However, $\gamma$ is not known. Therefore, the threshold model is estimated by ordinary least squares for all possible $\gamma$ values. For each $\gamma$, the Wald statistic of no difference between regimes is computed. To test the behavior of the threshold value, three statistics are computed: (i) sup-Wald, the maximum Wald value obtained over all simulations; (ii) avg-Wald, the average Wald value over all possible threshold values; (iii) exp-Wald, a function of the sum of exponential Wald statistics. An optimal threshold value is thus computed with an iterative procedure to separate the two regimes.

As suggested by Koop et al. (1996) and Pesaran and Shin (1997, 1998), we rely on the generalized impulse response function (GIRF) to identify the structural shocks in Eq. (23) since it is nonlinear*.* Indeed, GIRF provides a natural solution to the problems involved in defining impulse responses in nonlinear models (Franses and van Djick 2000). Note also that in a standard VAR, the intermediary shocks are null: only the shock at the impact differs from zero. The GIRF is not conditional on this assumption (Koop et al. 1996). More precisely, a linear model has a Wold representation in which shocks in different periods do not interact. However, nonlinear models cannot be rewritten using such a representation. They rather can be rewritten by means of the Volterra expansion in which shocks interact—the innovation at time *t* depends on past and future innovations. GIRF accounts for these interactions (Franses and van Dijk 2000). Finally, we use three lags for each variable to estimate the TVAR.

The Balke (2000) TVAR presents some advantages over the linear VAR model. Indeed, it accounts for two kinds of nonlinearities. First, in the linear VAR model, shocks are symmetric when they change sign in the sense that the responses to positive and negative shocks are mirror images of each other. In the TVAR, the impact of a shock depends on its sign: two shocks of equal magnitude have not necessarily the same impact in absolute value if their sign differs. Second, in the linear VAR model, the impulse response functions (IRFs) are proportional to the size of the shocks. If the shock is doubled, the amplitude of the IRF is also doubled. In contrast, the TVAR is not subject to this kind of constraint because changes in the IRFs may be non-proportional to the size of the shocks.^53^

### Results

#### GDP growth shocks

Figure 10 displays the plots of the impulse response functions (IRF) of the betas of the general index and strategies to the GDP growth shock using Balke’s (2000) algorithm.^54^ For the general index and each strategy, we distinguish a high and a low regime which is determined by GDP growth—i.e., the $\gamma$ statistic which is used to separate the two regimes. The computed optimal $\hat{\gamma }$ is close to 0, indicating that the low regime occurs when GDP growth is below 0% and the high regime sets in when GDP growth is higher than 0%. For the sake of parsimony, we do not report the Wald statistics associated with the cut-off value $\gamma$ but they indicate that this statistic is significant at the 5% level.^55^ For each regime, we have four IRFs. Indeed, shocks may be positive or negative. Moreover, we incorporate a moderate and a strong shock.

Before going further in our analysis, we sum up our results. First, the amplitude of the IRFs is generally higher in the low regime. Second, the response of the beta to a strong shock often varies proportionally more in the low regime, an obvious case of nonlinearity. However, some strategies seem to have difficulties in dealing with a strong shock in the low regime, which is less the case in the high one. Third, the behavior of the beta may be procyclical or countercyclical, and the degree of cyclicality may differ with the regime.

In the low regime, the response of the general index beta to a positive GDP growth shock is nonlinear, in the sense that the response to a strong shock is more than double the one associated with a moderate shock. Since the financial constraint is usually binding in the low regime, a strong shock may loosen this constraint quite substantially and provides hedge funds with more room of manoeuver to leverage (Eq. (5)). In the low regime, hedge funds decrease their beta following a moderate negative GDP shock—i.e., they deleverage—but the beta tends to increase temporarily when this shock is strong. Note that this behavior of the beta after a moderate shock is consistent with that obtained with LP. In this respect, the shocks generated by LP correspond to one standard deviation of the corresponding residual, which is the conventional way to devise shocks in VAR systems. This kind of shock corresponds to a moderate one in the TVAR approach, a strong shock being equal to two standard deviations of the corresponding residual.

The temporary increase in the beta of the general index after a strong shock suggests that hedge funds have difficulties in controlling a strong negative GDP shock at the impact in the low regime. In this respect, the response to a negative shock is much lower than the one associated with a positive one. In the high regime, the beta of the general index reacts more to negative than to positive shocks, which suggests that hedge funds better track their beta in this regime. However, the IRFs are not very sensitive to the size of the shock.

The beta of one important procyclical strategy—i.e., growth—responds substantially to a negative GDP shock in the low regime and this reaction is more than proportional to the size of the shock. This deleveraging (Eq. (5)) is therefore much more important with a strong shock than with a moderate one, which suggests good market timing. However, this strategy has more room of manoeuver to vary its beta in the high regime. For instance, the spreads in the amplitude of the IRFs between a strong and a moderate shock are greater in the high regime, which suggests once more a better control of market risk in this regime.

Some countercyclical strategies identified in the previous section—i.e., distressed, fixed income, and to a less extent convertibles and long-short credit—display a more significant reaction in the low regime. For instance, the betas of the distressed securities, long-short credit and fixed income strategies increase substantially in a nonlinear way following a strong negative GDP shock.^56^ This may be due to the convexity of bond prices with respect to yields, and to the nature of the business lines of these strategies. In this regard, the distressed securities strategy, which takes long positions in high-yield bonds and short positions in bonds with good credit ratings, may perform better during severe recessions when the probability of corporate bankruptcies peaks—i.e., the spreads between its long and short positions increase. To capture these spreads, this strategy may thus be induced to rise its systematic risk at that time by leveraging its beta. The case of the convertibles strategy is especially interesting since its beta is countercyclical in the high regime but procyclical in the low regime. Indeed, in this latter regime, its beta decreases after a negative GDP shock and the impact of a strong shock is proportionally greater than a moderate one. This behavior is related to the two dimensions of a convertible bond: (i) a bond; (ii) a stock, as embedded in its call option. In the low regime, the bond dimension dominates. A negative GDP shock tends to reduce the beta of the convertibles strategy since the call option incorporated in the convertible bond becomes more and more out-of-the-money with the size of the shock. Conversely, a positive GDP shock tends to increase the beta of convertibles bonds in the low regime, but this effect is very moderate. This explains the procyclicality of the beta of the convertibles strategy in the low regime. However, it becomes countercyclical in the high regime, while a convertible bond tends to behave as a stock. When a positive GDP shock occurs, the option value of a convertible bond increases, and this move tends to be more than proportional to the size of the shock. In this situation, the convertibles strategy partly hedges the equity risk of the hybrid securities it holds, which explains the decrease in its beta following a positive GDP shock which is more than proportional to the size of the shock (Fig. 10). In contrast, the option value of a convertible bond tends to decrease after a negative GDP shock. This fosters the convertibles strategy to reduce its hedging operations and tolerate a rise in its beta. The higher the size of the negative shock, the lower the increase in the beta is. These findings may explain why it was difficult to categorize the convertibles strategy as procyclical or countercyclical in "Stylized facts"  section..

The beta of the futures strategy displays a countercyclical pattern in both regimes, and it reacts more in a nonlinear way to negative than to positive GDP shocks. Following a negative GDP shock, its beta increases and this move is more than proportional to the size of the shock. This reaction does not signal inappropriate market timing. Indeed, the beta of the futures strategy tends to be negative during bad times, which is associated with a countercyclical behavior. This beta thus becomes less negative after an adverse GDP shock, a prudent move which allows benefiting from falling stock markets. However, consistent with our previous analysis of its cyclicality, the beta of the futures strategy tends to decrease after a positive GDP shock, albeit this change is very moderate. However, when the shock is large—especially in expansion—this strategy gives up this practice in order to take advantage from the upward trend in the stock market associated with a positive GDP shock. The beta of short-sellers is also countercyclical but behaves quite differently from the one of the futures strategy. It reacts much more to strong positive GDP shocks. In this regard, the beta of short-sellers jumps (in absolute value) at the impact after such shocks, and this behavior is more pronounced in the high regime. This shows the strategy is struggling to control big positive shocks, particularly during expansions when they are more frequent. Thereafter, its negative exposure to the stock market decreases, as it should be. However, consistent with their contrarian behavior, the beta of short-sellers increases (in absolute value) in both regimes following negative GDP shocks.

The response of the macro strategy to GDP shocks is also quite different from the others. In expansion, its beta is procyclical, although the amplitude of the IRFs is quite low. In the low regime, its beta is very sensitive to a positive shock. It increases following such a shock, and the response is more than proportional for a strong shock. This seems to be related to the high liquidity of this strategy’s portfolio (Table 2). The macro strategy also struggles to control a strong negative GDP shock in the low regime since its beta increases following the occurrence of this shock and remains practically unchanged after a moderate shock. The beta of the equity market neutral strategy is procyclical but it responds more to negative GDP shocks, especially in the high regime. Finally, the behavior of the beta of some strategies—i.e., mergers, multistrategy, opportunity index, and value index—is similar in the two regimes. Note that the betas of the mergers and opportunity index strategies display procyclicality according to TVAR even if it was difficult to categorize them in "Stylized facts"  section.

#### VIX shocks

We now investigate the response of our hedge fund indices to a VIX shock^57^ (Fig. 11). Similarly to our results with LP, we note that the general index responds much more to a VIX shock in the low regime regardless of the direction of the shock. For instance, following a positive (unfavourable) VIX shock, the IRF peaks at -0.02 for a moderate shock and at -0.04 for a strong shock. The representative hedge fund thus reduces its systematic risk when the volatility of financial markets is rising. However, the reaction of the beta to a VIX shock is greater when this shock is negative, which suggests that it is more difficult to track the beta for a positive VIX shock. In this respect, the corresponding IRF peaks at 0.03 for a moderate negative shock and at 0.06 for a strong shock. Importantly, analogously to the LP method, the beta of the general index is more sensitive to a VIX shock than to a GDP growth one, regardless of the regime.

Strategies with the highest betas—i.e., growth (0.84), value index (0.59), opportunity index (0.47), event-driven (0.43), and multistrategy (0.40)—are the most responsive to VIX shocks. Their responses are similar to the one of the general index. They are much more important during the low regime regardless of the sign or the size of the shocks—an obvious source of asymmetry—and the reaction is greater for negative than for positive shocks. For instance, in the low regime, the IRF of the growth strategy’s beta peaks at 0.055 for a moderate negative VIX shock and at 0.13 for a strong one. The response of the beta of the growth strategy is clearly non-proportional to the size of the shock. Turning to positive VIX shocks, the IRF of this strategy peaks at -0.05 for a moderate shock and at -0.10 for a strong one. This means the response is greater for negative than for positive shocks. It appears more difficult to track positive than negative shocks. Moreover, a strong negative shock may loosen the liquidity constraint and foster leveraging which is less the case for a moderate shock. This can explain the non-proportional response of the beta of the growth strategy to negative VIX shocks. These shocks could thus be mainly responsible for the volatility of procyclical strategies’ betas. Even if they have a smaller beta, the plots of the IRFs of the mergers, equity market neutral and convertibles strategies are similar to those of the most procyclical strategies. In this respect, analogously to our LP analysis, the beta of the equity market neutral strategy is much more sensitive to VIX shocks in the low than in the high regime. Still in line with our findings with LP, in the low regime, after a positive VIX shock, the convertibles strategy quickly hedges the equity risk associated with the hybrid securities it holds, so its beta decreases. When a negative VIX shock occurs, it relaxes its hedging operations, so its beta increases. The beta response to a strong negative shock is more than proportional to a moderate one since the value of the call option incorporated in a convertible bond impacts the beta in a nonlinear way.

Macro is another strategy which displays an atypical behavior in the low regime. In the high regime, its beta is procyclical. However, in the low regime, this strategy has difficulties in reducing its beta after a positive VIX shock. According to the IRFs, it struggles to hold back the rise in its beta in this regime. Once more, this fact may be explained by the high level of liquidity of its portfolio (Table 2). Similarly to its pattern associated with a positive GDP shock, the beta of the macro strategy is very sensitive to a negative VIX shock in the low regime and its response to a strong shock is more than proportional to a moderate one. Indeed, its beta registers a moderate jump after a strong negative shock which is much higher than the corresponding increase in the high regime.

Short-sellers and futures strategies respond once again very differently to VIX shocks. Since short-sellers are involved in contrarian activities relatively to procyclical strategies, they obviously benefit from positive VIX shocks, which are associated with falling stock markets. Analogously to our results with LP, they increase their negative exposure to stock markets in order to leverage their returns. Not surprisingly, their response is much more important in the low regime. In this respect, the IRF peaks at -0.1 for a moderate positive shock and at -0.3 for a strong shock. The futures strategy—that is also quite involved in short sales—displays a contrarian reaction to VIX shocks in the low regime while the amplitude of its IRFs is much lower in the high regime. In the low regime, after a positive VIX shock, its beta increases, but since it is then negative, it decreases in absolute value. Similarly to its behavior after a negative GDP shock, it implements a prudent approach to generate positive payoffs. In contrast, after a negative VIX shock, its beta decreases, so it seems to hedge market risk in this situation. The beta of the futures strategy thus tends to follow a mean-reverting process. The fact that its average beta is close to zero over our sample period supports this conjecture (Table 2). Its beta is much less sensitive to VIX shocks than the one of short-sellers who maintain their beta in the negative range.

The behavior of countercyclical strategies—i.e., fixed income, distressed and to a less extent long-short credit—still differs from the others in the low regime. The fixed income strategy has difficulties in controlling positive VIX shocks—especially big ones—in the low regime. Indeed, its beta may increase for several quarters. However, its beta displays a substantial increase after a strong negative VIX shock, this strategy seeking to capture the credit risk premium by bearing more risk. Indeed, this strategy seeks to grip this premium by being long on high yield bonds, which are quite risky, and short on bonds with a good credit rating. The return spread between these two kinds of bonds usually peaks after a jump in the VIX, which might explain the behavior of the beta of the fixed income strategy. The distressed securities strategy is at play in the low regime: it seems to benefit from business opportunities after positive VIX shocks. Indeed, its beta increases after some quarters—especially for strong shocks— and persists on this upward trend, which suggests that this strategy increases its risk to benefit from the deterioration of firms’ financial health associated with falling markets.^58^ The distressed securities strategy also seizes the opportunity provided by negative VIX shocks to leverage its beta but the upward trend of the IRF is less persistent, negative VIX shocks tending to occur near the end of a low regime when the business perspectives improve. In this regime, the long-short credit strategy also adopts a similar approach following a strong positive VIX shock but with more prudence than the distressed strategy. Indeed, its long-short transactions which resort to credit derivatives moderate the rise in its beta. In order to seize the risk premia which are higher in the low regime, this strategy fosters a higher increase in its beta for negative VIX shocks—especially strong shocks—and the IRF is more persistent. Actually, even if risk premia may be lower when VIX shocks are negative, they still remain substantial in the low regime due to the prevalence of a high level of uncertainty which is much less important in the high regime.

Overall, the results obtained with TVAR are consistent with those obtained with LP in the low regime for a VIX shock albeit we note some differences due to the severity of the subprime crisis.

#### Liquidity shocks

Figure 12 provides the IRFs associated with TVAR computed with *i_gamma* and $\widehat{cv\_igamma}$ shocks for the general index, and the growth and fixed income strategies. As usual, the threshold is determined by real GDP growth. In the high regime, we note that the general index beta decreases when *i_gamma* decreases^59^ and, conversely, the beta increases when *i_gamma* trends upward. The responses are quite proportional to the size of the shocks. Hedge funds thus seem to manage their illiquidity risk in the high regime. They reduce their systematic risk when illiquidity risk is oriented upward but they have more room of manoeuver to increase their systematic risk when illiquidity risk trends downwards. The response of the beta to *cv_igamma* is much less important, uncertainty related to liquidity being low in the high regime. For instance, the rise in the beta is higher than its initial decrease after a positive *cv_igamma* shock, suggesting that hedge funds seek to capture the illiquidity premium. When *cv_igamma* decreases, there is a drop in the beta which is much more important than the previous initial rise, suggesting that hedge funds take advantage of the decrease in illiquidity uncertainty to reduce their systematic risk. This response in more than proportional with a big shock. Hence, in the high regime, hedge funds react quite differently to *i_gamma* and *cv_igamma* shocks.

In the low regime, the response of the general index beta to an upside and downside *i_gamma* shock is less than in the high regime. Indeed, consistent with the results obtained with LP, the beta also decreases after a negative shock but less than in the high regime. Hence, hedge funds seem to struggle to control their beta in the low regime. Following a positive *i_gamma* shock, the beta increases more than it decreases after a negative shock. The response to a big positive shock is more than proportional, suggesting that the shock must be large enough in order to foster the representative hedge fund to take more risk in the low regime. In contrast to *i_gamma*, in line with our findings with LP, the response of the hedge fund beta to *cv_igamma* is much higher in the low regime. This is an obvious asymmetry in the behavior of hedge funds according to the phase of the business cycle. Following an unfavourable *cv_igamma* shock, hedge funds reduce their beta—i.e., they deleverage—and the impact of a big shock is more than proportional. Therefore, hedge funds respond very significantly to this kind of uncertainty shock. When the *cv_igamma* shock is negative, hedge funds increase their beta. However, a big shock has a less than proportional impact on the beta, signalling that hedge funds are reluctant to increase their beta following a decrease in liquidity uncertainty in the low regime. They thus adopt a prudent behavior.

Among strategies studied in our paper, the growth strategy exhibits the highest beta. It is therefore normal that this strategy responds more to shocks than the representative hedge fund, as measured by the behavior of the general index beta. In the high regime, the reaction of its beta to *i_gamma* shocks has a pattern similar to the general index although it is more cyclical. However, the dynamics of the growth strategy’s beta after a *cv_igamma* shock is quite different from an *i_gamma* shock. In the high regime, it increases following a positive *cv_igamma* shock, suggesting that the growth strategy seeks to capture the illiquidity premium when uncertainty related to illiquidity trends downward. Note that the responses to big and moderate shocks are quite close. The growth strategy thus remains prudent when seeking to capture the illiquidity premium. In the low regime, the response of the growth strategy’s beta to *i_gamma* shocks is higher. For instance, the impact of a big negative shock on its beta is twice the one observed in the high regime. The growth strategy thus reduces substantially its beta when confronted to a rise in liquidity risk. The reverse is also true for a reduction in this kind of risk. The response of its beta to *cv_igamma* is however less in the low regime. At the impact of an increase in this shock, the beta decreases but, with a delay of three quarters, it overshoots upside, suggesting that the growth strategy still seeks to capture the illiquidity premium.

In the high regime, the response of the fixed income strategy to *i_gamma* and *cv_igamma* shocks shows that it controls quite well its systematic risk—i.e., its beta decreases for unfavourable shocks and increases for favourable ones—and the amplitude of the IRFs is quite high given the low market beta of this strategy. However, the picture changes drastically in the low regime. The beta increases after an unfavourable *i_gamma* shock and the amplitude of the IRF of a big shock is four times the one of a moderate shock. The fixed income strategy thus struggles to control its liquidity risk in the low regime—especially risk stemming from big shocks. We note the same difficulties on the side of *cv_igamma* when a positive shock occurs—i.e., when illiquidity uncertainty increases. The fixed income strategy succeeds in reducing its beta for a moderate shock while a big shock appears less manageable.

## Appendix 2

### The impact of the Amihud ratio on strategies’ returns and conditional betas

In this appendix, analogously to Tables 4 and 5 built with *IML*, we resort to Eq. (18) to estimate the impact of the Amihud (2002) ratio on hedge fund strategies’ returns and betas using, as for the previous tables, a pooled regression with SUR built on strategies.

The Amihud illiquidity measure is the daily ratio of absolute stock returns scaled by dollar volume, averaged over each month, i.e., $Amihud = \frac{1}{{D_{i} }}\sum\nolimits_{i = 1}^{{D_{i} }} {\frac{{\left| {R_{i} } \right|}}{{Vol_{i} }}}$, where *D*_*i*_ is the number of days in the month, *R*_*i*_ is the daily return on stock *i* and *Vol*_*i*_ is its corresponding trading volume. We compute the Amihud (2002) illiquidity measure with the S&P500. This ratio is detrended using the Hodrick-Prescott filter as in Naes et al. (2011). The Amihud ratio quantifies the price/return response to a given size of trade. When the Amihud ratio is high, liquidity is low.

Table

**Table 6:** Sensitivities of hedge fund strategies’ returns to the Amihud ratio using pooled regressions with fixed coefficients and SUR, 1995–2016

	Amihud				
	Crises	Outside crises	Subprime	Outside subprime	Whole sample
General index	− 0.10	0.16	0.95	0.24	0.18
	− *0.28*	*0.54*	*1.52*	*1.00*	*0.79*
Convertibles	0.56	0.31	0.47	**0.75**	**0.55**
	*1.05*	*0.67*	*0.55*	***2.23***	***1.62***
Distressed securities	0.25	0.42	0.44	**0.68**	**0.48**
	*0.66*	*1.33*	*0.71*	***2.80***	***1.97***
Event driven	− 0.55	0.18	0.80	0.06	0.01
	− *1.44*	*0.58*	*1.26*	*0.23*	*0.02*
Equity market neutral	**0.77**	− 0.26	**1.70**	0.28	**0.32**
	***2.35***	− *0.97*	***3.09***	*1.30*	***1.60***
Fixed income	0.47	− 0.06	0.71	**0.42**	0.30
	*1.23*	− *0.19*	*1.15*	***1.76***	*1.31*
Futures	1.21	**1.34**	1.60	**1.58**	**1.42**
	*1.30*	***1.67***	*1.01*	***2.47***	***2.14***
Growth	− **2.17**	0.40	0.33	− 0.53	− 0.57
	− ***2.57***	*0.55*	*0.22*	− *0.87*	− *1.00*
Long-short credit	**0.94**	− 0.24	**1.64**	**0.38**	**0.40**
	***2.77***	− *0.88*	***2.95***	***1.79***	***1.92***
Macro	− 0.20	− 0.18	0.97	− 0.03	− 0.05
	− *0.38*	− *0.39*	*1.03*	− *0.10*	− *0.15*
Mergers	0.13	− 0.06	**1.08**	− 0.09	0.02
	*0.40*	− *0.16*	***2.55***	− *0.57*	*0.14*
Multistrategy	− 0.05	− 0.22	**1.35**	− 0.05	− 0.01
	− *0.13*	− *0.70*	***2.00***	− *0.19*	− *0.05*
Opportunity index	− 0.63	0.38	0.55	0.18	0.08
	− *1.19*	*0.84*	*0.59*	*0.50*	*0.22*
Short-sellers	**3.15**	− 0.65	1.65	1.23	1.13
	***1.76***	− *0.41*	*0.54*	*0.99*	*0.98*
Value index	− **1.80**	− 0.54	− **2.16**	− 0.56	− **0.95**
	− ***2.56***	− *0.88*	− ***1.77***	− *1.13*	− ***2.04***
All strategies	0.17	0.03	**1.00**	**0.30**	**0.20**
	*0.68*	*0.13*	***2.40***	***1.97***	***2.09***

This table provides the estimation of Eq. (18) using pooled regressions accounting for the interaction between strategies’ innovations (SUR, i.e., seemingly unrelated regressions, Zellner 1962). Eq. (18) is estimated over the whole sample period and over our two scenarios described in "Robustness check: response of hedge fund strategies to illiquidity shocks" section. In order to avoid overloading this table, we only report the estimated coefficients associated with the Amihud (2002, 2019) ratio for each strategy. t-statistics are in italics. Shaded coefficients are significant at the 10% level or less

6, which corresponds to Table 4 for *IML*, reports the loadings of the Amihud ratio for hedge fund strategies’ returns. This ratio impacts less hedge fund returns than *IML*. Moreover, the direction of the impact may differ substantially from the one observed with *IML*. Indeed, in contrast to *IML*, the returns of the growth and value index strategies respond negatively and significantly to the Amihud ratio during crises. As contended in "Impact of IML on strategies’ returns", section the uncertainty effect related to the illiquidity ratio may explain this reaction—i.e., a negative sign for the coefficients of the illiquidity measures in cross-section return equations (Acharya and Pedersen 2005; Konstantopoulos 2016). The Amihud ratio, which co-moves more with stock market volatility than *IML*, might be more related to the “uncertainty effect”. Strategies having a higher exposure to market systematic risk should be more subject to this effect. Conversely, we observe that strategies having a low or negative conditional beta—i.e., equity market neutral, long-short credit, and short-sellers—respond positively and significantly to the Amihud ratio during crises. We note a similar pattern over the subprime crisis. During the whole sample period, note that strategies which are particularly illiquid—i.e., convertibles, distressed, and long-short credit—display, as expected, a positive and significant response to the Amihud ratio. It is thus easier to capture the illiquidity premium associated with these strategies over the long-run.

Consistent with the results obtained with the *IML* illiquidity measure (Table 5), the conditional beta of hedge fund strategies tends to respond negatively to the Amihud ratio (Table

**Table 7:** Sensitivities of hedge fund strategies’ betas to the Amihud ratio using pooled regressions with fixed coefficients and SUR, 1995–2016

	Amihud				
	Crises	Outside crises	Subprime	Outside subprime	Whole sample
General index	− **0.03**	− **0.02**	**0.06**	− **0.03**	− **0.03**
	− ***2.39***	− ***2.29***	***2.44***	− ***3.85***	− ***3.37***
Convertibles	− 0.04	− 0.66	**0.11**	− **0.04**	− 0.03
	− *1.13*	− *0.02*	***2.14***	− ***2.01***	− *1.33*
Distressed securities	− **0.13**	− **0.02**	0.01	− **0.08**	− **0.08**
	− ***7.92***	− ***2.04***	*0.02*	− ***5.90***	− ***5.78***
Event driven	− **0.18**	− **0.04**	0.01	− **0.11**	− **0.11**
	− ***6.83***	− ***3.13***	*0.17*	− ***8.91***	− ***8.21***
Equity market neutral	− **0.17**	− **0.04**	− 0.02	− **0.11**	− **0.11**
	− ***8.29***	− ***2.85***	− *0.62*	− ***8.05***	− ***7.88***
Fixed income	− **0.02**	− 0.01	**0.07**	− **0.02**	− 0.01
	− ***1.96***	− *0.04*	***3.45***	− ***2.26***	− *1.56*
Futures	− **0.23**	− 0.02	− 0.02	− **0.13**	− **0.12**
	− ***1.68***	− *0.75*	− *0.32*	− ***5.13***	− ***5.06***
Growth	− **0.10**	0.01	**0.18**	− **0.07**	− 0.04
	− ***1.89***	*0.28*	***1.83***	− ***1.70***	− *1.07*
Long-short credit	− **0.14**	− 0.01	0.01	− **0.07**	− **0.07**
	− ***8.03***	− *0.23*	*0.34*	− ***6.52***	− ***6.27***
Macro	− 0.01	0.01	0.09	− 0.01	− 0.01
	− *0.25*	*0.18*	*1.06*	− *0.32*	− *0.13*
Mergers	− **0.16**	0.01	− **0.11**	− **0.06**	− **0.07**
	− ***8.60***	*0.62*	− ***3.13***	− ***4.38***	− ***5.19***
Multistrategy	0.03	**0.13**	0.08	**0.09**	**0.08**
	*1.49*	***4.47***	*1.21*	***3.87***	***3.71***
Opportunity index	− **0.15**	− 0.02	0.11	− **0.11**	− **0.08**
	− ***3.98***	− *0.68*	*1.44*	− ***3.33***	− ***2.75***
Short-sellers	− **0.24**	− **0.23**	− 0.19	− **0.24**	**0.07**
	− ***2.72***	− ***3.12***	− *1.05*	− ***3.05***	***1.66***
Value index	0.09	0.07	**0.39**	0.03	0.13
	*1.36*	*1.23*	***3.29***	*0.68*	*0.56*
All strategies	− **0.05**	0.01	**0.04**	− **0.05**	− **0.02**
	− ***5.28***	*0.01*	***3.46***	− ***12.64***	− ***5.69***

This table provides the estimation of Eq. (18) using pooled regressions accounting for the interaction between strategies’ innovations (SUR, i.e., seemingly unrelated regressions, Zellner 1962). Eq. (18) is estimated over the whole sample period and over our two scenarios described in "Robustness check: response of hedge fund strategies to illiquidity shocks" section. In order to avoid overloading this table, we only report the estimated coefficients associated with the Amihud (2002, 2019) ratio for each strategy. t-statistics are in italics. Shaded coefficients are significant at the 10% level or less

7). For instance, without cross-section specific effects, the coefficient associated with the Amihud ratio is equal to -0.05 during crises, significant at the 1% level. Only four strategies—i.e., convertibles, macro, multistrategy, and value index—do not respond significantly to this ratio during crises. The beta of the other strategies displays a significant negative response which is more important than over the whole sample period, suggesting that asymmetries are at play during crises. Strategies whose beta responds the most seem to be the ones whose activities are particularly impaired by a rise in illiquidity—i.e., short-sellers, futures, event-driven, equity market neutral and mergers. For these strategies, the “uncertainty effect” associated with the Amihud ratio should also be at play.

Interestingly, the beta of fewer strategies responds to the Amihud ratio during the subprime crisis. In contrast to the scenario melting all crises, the reaction tends to be positive. For instance, the value index and growth strategies have the highest loadings to the Amihud ratio, 0.39 and 0.18, respectively. We may conjecture that these strategies sought to capture the illiquidity premium during the subprime crisis. For the fixed income and convertibles strategies, the increase in the beta may rather be associated with their difficulties in controlling systematic risk during the crisis. In common with the scenario combining all crises, the beta of the mergers strategy has trended downwards during the subprime crisis, suggesting that illiquidity is a real impediment for this strategy.

## Footnotes

1 Note that the hedge fund industry is also relatively hermetic compared to other financial institutions like banks. Improving the quality of information in this industry might reduce stock price crash risk (Wen et al. 2019). This is a research avenue in the hedge fund field.

2 As discussed in “Policy implications” section, another motivation of our article is to propose risk indicators and tools which may prove useful to conduct the resilience tests required by the new hedge fund legislation in the European Union.

3 In “Appendix 2”, we complete this analysis by examining the impact of another well-known illiquidity measure—i.e., the Amihud (2002) ratio—on hedge fund returns and conditional betas.

4 In financial economics, absolute risk aversion is defined as $- \frac{u^{\prime\prime}}{{u^{\prime}}}$, where *u* is the utility of an agent defined as a function of wealth and with $u^{\prime\prime} < 0$. Absolute risk prudence is defined as $- \frac{u^{\prime\prime\prime}}{{u^{\prime\prime}}}$ with $u^{\prime\prime\prime} > 0$ (e.g., Bertrand and Prigent 2010).

5 Note that when using Balke’s (2000) procedure, the number of shocks is usually restricted to one or two because it is very time-consuming. Moreover, running this procedure with too many shocks may destabilize it substantially since it requires the estimation of too many parameters.

6 The LP method is concerned with moderate shocks, and not strong shocks.

7 i.e., the response is more than proportional to the size of the shock.

8 Note that market timing by hedge funds will surely benefit in the future from the explosion of FinTech platforms in terms of quantitative research and financial information sharing. On this issue see: Arslanian, H., 5 ways FinTech will change the hedge fund industry (www.henriarslanian.com); Broby (2021); and Kou et al. (2021).

9 However, this method presents some econometric problems like errors-in-variables. See Racicot et al. (2019).

10 A fairly close econometric method to make coefficients time-varying is the rolling regression. In this respect, Ghysels and Marcellino (chap. 11, 2018) compare the Kalman filter to the rolling regression and they find that the former is not significantly better than the latter. For a comparison of these two methods using hedge fund data, see Racicot et al. (2019).

11 See, for instance, Lambert and Plantania (2020) and Racicot et al. (2021) for a discussion on the different responses of hedge fund strategies to shocks.

12 GAI’s database website is: http://www.greenwichai.com.

13 The span of our sample is more important than the number of observations per se as it covers several business cycles (Shiller and Perron 1985).

14 Source: Greenwich Alternative Investment website (2016).

15 Another bias is the backfill one or the instant history bias. An increase in the incubation period—i.e., the time between inception and the listing in a database—leads to a higher backfill bias.

16 For instance, Lambert and Plantania (2020) rely on the Kalman filter to compute the time-varying alphas and betas associated with their hedge fund database.

17 In fact, the link between the beta of a firm and its leverage is a synthesis of the corporate finance theory developed by Modigliani and Miller (1958, 1963) and the CAPM asset pricing model. This synthesis was provided by Rubinstein (1973).

18 See "Policy implications" section.

19 As contended by De Groot and Franses (2008), economic and financial variables may display multiple cycles of different periods. They refer to the three to four year Kitchin inventory cycle, the 7–11 year Juglar cycle, the 15–25 year Kuznets cycle, and the 48–60 year Kondratieff cycle. The first one is associated with firms’ inventories, the second with investment in machines, the third with migration and investment in construction, and the later with structural economic development. Analogously, the hedge fund beta may display multiple cycles, an interesting issue which is beyond the scope of our paper.

20 $$d\ln GDP_{t} = \ln \left( {\frac{{GDP_{t} }}{{GDP_{t - 1} }}} \right)$$.

21 When the beta of a strategy is positive, it is viewed as countercyclical when the sum of lags associated with this strategy is negative, and vice-versa.

22 Note that a VAR model ought to be parsimonious since it consumes many degrees of freedom due to the fact that variables are lagged over some quarters.

23 The AIC_c_ is a corrected version of the AIC criterion proposed by Hurvich and Tsai (1993) and specifically designed for VARs. See also Jordà (2005).

24 Note that Fig. 1 is built with positive GDP shocks. Since we consider negative shocks in this section, we discuss the mirror images of the plots included in Fig. 1, since positive and negative shocks are symmetric for classical linear VAR and linear LP models.

25 They increased their exposure to the VIX since a rise in this indicator is associated with falling stock markets which provide positive payoffs to contrarian strategies.

26 Regarding the cyclical dimensions of the term spread and the credit spread, see Naes et al. (2011). Similarly to the Amihud illiquidity ratio, these authors also find that these variables are very good leading indicators of economic activity.

27 “Firm” can refer here to a financial institution such as hedge funds.

28 The Fama and French factors appearing in our pooled regressions are drawn from the French’s website (https://mba.tuck.dartmouth.edu/pages/faculty/ken.french/data_library.html). The financial variables are drawn from the Fred database (https://fred.stlouisfed.org/). The *IML* illiquidity measure comes from the Pastor’s database (https://faculty.chicagobooth.edu/lubos.pastor/research/).

29 The impact of the Amihud ratio on the hedge fund conditional beta is analyzed in “Appendix 2”.

30 See Fung and Hsieh (2004) for the presence of the term spread, the credit spread and the first-difference in the ten-year rate in the hedge fund asset pricing equation, and Racicot et al. (2019) for the presence of *IML* and the Amihud ratio. Note that we rely on an approach that differs from these authors. Moreover, in contrast to Fung and Hsieh (2004), we exclude lookback straddles in Eq. (18)for the sake of parsimony but also because they are mainly related to the volatility of financial markets.

31 Note that we exclude the VIX from this equation due to the interaction between illiquidity measures and VIX. Moreover, as noted previously, the term spread is also related to market illiquidity.

32 Recall that the CAPM and its extensions are concerned with expected returns while the market model deals with contemporaneous returns (observed returns).

33 See also Lambert and Plantania (2016, 2020).

34 Which corresponds to the “all strategies” line in Table 4.

35 See "Positive VIX shocks" section.

36 See “Appendix 1” for the corresponding computations with TVAR.

37 These computations are available upon request.

38 Or TVAR in “Appendix 1”.

39 As opposed to *IML* which is an illiquidity measure.

40 One may object that the *i_gamma* measure of market liquidity is also a generated variable since it is devised using a regression. However, Pástor and Stambaugh (2003, p. 650) are aware of the potential bias associated with generated variables. They contend that since their liquidity indicator is an average of a very large number of stocks, the measurement-error bias is thus minimized.

41 On the issue of generated variables, see also: Beaudry et al. (2001), Baum et al. (2009), Calmès and Théoret (2014), Racicot and Théoret, (2016).

42 The computation of the Amihud ratio is explained in “Appendix 2”.

43 According to this correlation, the returns of illiquid stocks decreased markedly during the subprime crisis.

44 Recall that the beta of the growth strategy is more procyclical than the one of the general index.

45 Note that there are other tools of “quantitative easing” at the disposal of a central bank like the purchase of non-traditional assets such as MBS.

46 On the fire-sales externality, see also Stein (2009).

47 See also Stafylas et al. (2017).

48 Recall that the local projection is performed using a negative i_gamma shock—i.e., an illiquidity shock.

49 On this issue, see *Hedgeweek,* April 22, 2020. Note that the total AUM of hedge funds recovered thereafter and soared to $US 3.8 trillion in the first quarter of 2021 and that this quarter has been the strongest in terms of profits since 2000 (Hedgeweek, April 21, 2021).

50 For more detail see Xavier Parain, *Hedgeweek*, July 15, 2020.

51 This analysis appears in “Appendix 1”.

52 For instance, in the deleveraging cycles of Brunnermeier and Pedersen (2009), an increase in market volatility results in an increase in margins which tend to reduce leverage (Ang et al. 2011).
